# Foxa2 identifies a cardiac progenitor population with ventricular differentiation potential

**DOI:** 10.1038/ncomms14428

**Published:** 2017-02-14

**Authors:** Evan Bardot, Damelys Calderon, Francis Santoriello, Songyan Han, Kakit Cheung, Bharati Jadhav, Ingo Burtscher, Stanley Artap, Rajan Jain, Jonathan Epstein, Heiko Lickert, Valerie Gouon-Evans, Andrew J. Sharp, Nicole C. Dubois

**Affiliations:** 1 Department of Cell, Developmental and Regenerative Biology, Icahn School of Medicine at Mount Sinai, Box 1040, 1470 Madison Avenue, New York, New York 10029, USA; 2 Mindich Child Health and Development Institute, Icahn School of Medicine at Mount Sinai, New York, New York 10029, USA; 3 Black Family Stem Cell Institute, Icahn School of Medicine at Mount Sinai, New York, New York 10029, USA; 4 Department of Genetics and Genomic Sciences, Icahn School of Medicine at Mount Sinai, New York, New York 10029, USA; 5 Institute of Diabetes and Regeneration Research at the Helmholtz Zentrum München, German Center for Diabetes Research (DZD), Neuherberg 85764, Germany; 6 Departments of Cell and Developmental Biology and Medicine, Cardiovascular Institute, University of Pennsylvania, Philadelphia, Pennsylvania 19104, USA

## Abstract

The recent identification of progenitor populations that contribute to the developing heart in a distinct spatial and temporal manner has fundamentally improved our understanding of cardiac development. However, the mechanisms that direct atrial versus ventricular specification remain largely unknown. Here we report the identification of a progenitor population that gives rise primarily to cardiovascular cells of the ventricles and only to few atrial cells (<5%) of the differentiated heart. These progenitors are specified during gastrulation, when they transiently express *Foxa2*, a gene not previously implicated in cardiac development. Importantly, *Foxa2+* cells contribute to previously identified progenitor populations in a defined pattern and ratio. Lastly, we describe an analogous Foxa2+ population during differentiation of embryonic stem cells. Together, these findings provide insight into the developmental origin of ventricular and atrial cells, and may lead to the establishment of new strategies for generating chamber-specific cell types from pluripotent stem cells.

## Abstract (web summary)

The progenitor populations that contribute to the key cardiac lineages in a chamber-specific manner are unknown. Here, the authors identify *Foxa2*+ progenitor population, which is specified at gastrulation, as contributing primarily to cardiovascular cells of both ventricles and the epicardium in mice.

Heart development is a complex morphogenetic process that integrates cells from multiple origins into a well-organized structure^1^,^2^. The correct specification of progenitor populations and the ability of these cells to respond to spatiotemporal cues in the early embryo are critical for successful formation of the four-chambered heart. Errors in this process can result in congenital heart defects, which affect about 8 in 1,000 newborns and over 1 million adults in the United States alone^3^,^4^,^5^,^6^. Although treatments for some of these defects have improved, the underlying causes remain poorly understood. Increasing our understanding of the morphogenesis and cellular origin of the heart will advance our knowledge of how heart defects occur, as well as provide an important framework for designing better *in vitro* models and regenerative approaches using pluripotent stem cell (PSC) systems.

Research in mouse, chick and zebrafish has identified four main progenitor populations that give rise to distinct areas of the mature heart: the first and second heart fields (FHF and SHF), neural crest cells, and the proepicardial organ (PEO)^2^,^7^,^8^,^9^,^10^,^11^. The FHF and SHF give rise to the majority of the heart, with the left ventricle, most of the atria and part of the right ventricle (RV) deriving from the FHF, and the SHF giving rise to the RV, outflow tract and part of the atria. Interestingly, these progenitor pools are defined by their spatial arrangement during heart tube morphogenesis rather than by their specific cell fate potential. None of these progenitor populations contributes to the heart in a strictly chamber-specific manner. The distinct morphological and functional differences between atrial and ventricular cardiomyocytes raise the question as to whether chamber-specific progenitor populations exist before atrial and ventricular differentiation. However, the lack of tools and paucity of molecular information has thus far not allowed for a definitive confirmation of this intriguing hypothesis, nor for the isolation and detailed characterization of atrial and ventricular progenitor populations and the underlying mechanisms regulating their specification.

Several selective fate-mapping studies have identified cardiac progenitors at the gastrulation stage, further implying early specification of the cardiac lineages^12^,^13^,^14^,^15^. These studies suggest that the cardiac mesoderm (CM) is formed from the posterior epiblast and migrates anteriorly, where it takes up residence beneath the head folds to form the cardiac crescent (CC). Retrospective clonal analysis revealed common origins for myocardial cells in multiple regions of the developing heart, supporting the proposed early emergence of specified cardiac progenitors^9^,^16^,^17^. Explant experiments in the avian model system and fate mapping in the fish have suggested that atrial and ventricular cells are specified during gastrulation and defined by their anteroposterior position in the primitive streak (PS)^14^,^18^,^19^. More recent lineage tracing experiments using *Mesp1* or regulatory regions for *Smarcd3* identified cells during gastrulation that have already acquired a cardiac fate^20^,^21^. Although these efforts have greatly advanced our knowledge of the timing of heart specification, many open questions remain with respect to the mechanistic regulation of these early migration and specification events.

In this study, we provide evidence that transient expression of *Foxa2* during gastrulation labels a progenitor population that gives rise selectively to cardiovascular cells of both ventricles, as well as to epicardial cells. As such, *Foxa2* lineage tracing enables the visualization and isolation of prospective ventricular cells throughout development. Analysis at the CC stage shortly before cardiac differentiation and formation of the primitive heart tube (PHT) reveals *Foxa2* lineage-traced ventricular progenitors in a distinct localization that overlaps with both FHF and SHF cells. *Foxa2* is a pioneer transcription factor active during gastrulation and previously implicated primarily in endoderm and ectoderm lineage specification^22^,^23^,^24^, but not in heart development. Using mixed chimera experiments we show that Foxa2 seems to be required for the normal development of ventricular cells. Finally, translation of our findings to the mouse embryonic stem cell (mESC) system identified an analogous Foxa2-derived CM population during differentiation to cardiomyocytes and demonstrates reduced cardiac differentiation potential of Foxa2-deficient embryonic stem cells (mESCs). Our data provide an approach to genetically label a ventricular cardiac progenitor population as early as during gastrulation, enabling the isolation and characterization of prospective ventricular cells at any stage during development.

## Results

### Foxa2+ cells at gastrulation give rise to cardiac ventricles

**Figure 1:**
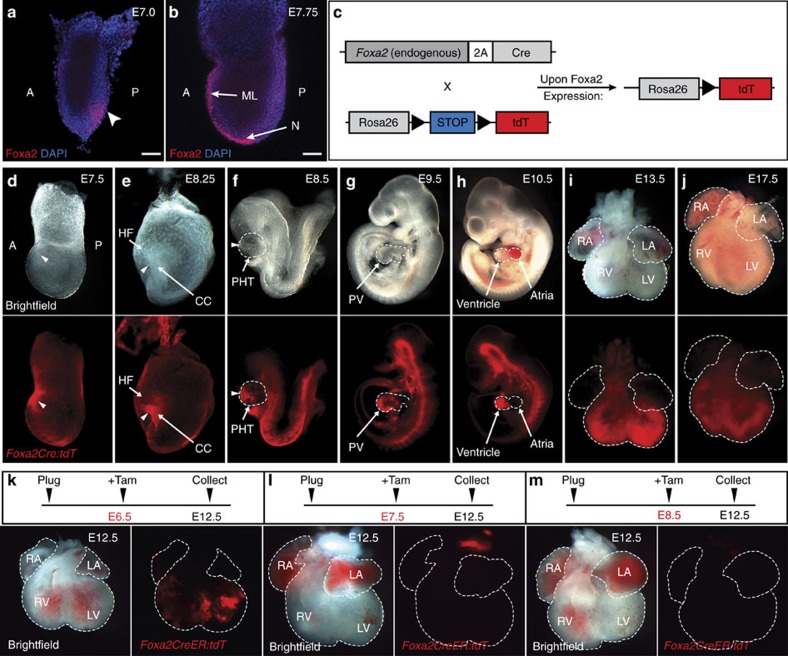
Lineage tracing of Foxa2-expressing cells during mouse development. (**a**,**b**) Representative confocal images of WMIF of E7.0 (**a**) and E7.75 (**b**) embryos with antibodies against Foxa2. Embryos were counterstained with 4,6-diamidino-2-phenylindole (DAPI) to label nuclei. Arrowhead shows the presumptive cardiogenic region of the PS. Scale bars, 75 μm. (**c**) Schematic of the *Foxa2* lineage-tracing system that results in permanent labelling of all cells that express *Foxa2* during development. (**d**–**j**) Whole-mount imaging of *Foxa2Cre* lineage-tracing embryos (**d**–**h**) or dissected hearts (**i**,**j**). *Foxa2Cre* embryos crossed with *R26-tdT* show labelling of the ventricular portion of the developing heart tube and heart. (**k**–**m**) Whole-mount imaging of dissected hearts of *Foxa2CreER*;*tdT* embryos. Tam (0.05 mg g^−1^) was injected at E6.5 (**k**), E7.5 (**l**) or E8.5 (**m**) and embryos were collected at E12.5. Dashed lines outline the heart tube (**f**–**h**) or the heart chambers (**i**–**m**). A, anterior; CC, cardiac crescent; HF, head folds; LA, left atria; LV, left ventricle; ML, midline; N, node; P, posterior; PHT, primitive heart tube; PV, primitive ventricle; RA, right atria; RV, right ventricle.

Long-standing evidence and recent work both suggest that cardiac specification events occur before the establishment of the PHT during mammalian development^14^,^19^,^20^,^21^,^25^,^26^. However, the key regulators of these cell-fate specification events have been challenging to study, largely due to the inability to prospectively identify early chamber-specific progenitor populations. To identify such cardiac progenitor populations during early embryogenesis, we turned our attention to genes that are known to be critical for cell-fate specification events during gastrulation and that are expressed in the prospective mesoderm in the PS. An analysis of early expression patterns revealed that the pioneer transcription factor *Foxa2* is present in the epiblast underlying the anterior PS region (Fig. 1a). Importantly, as *Foxa2* is an organizer gene, its expression domain overlaps with the areas derived from the organizer, including CM^27^. As the embryo develops, Foxa2 protein is rapidly downregulated in the anterior PS and is restricted to the node, midline and endoderm, as expected (Fig. 1b). To investigate whether Foxa2-expressing cells will contribute to cardiac structures later during development, we used a recently established *Foxa2* lineage-tracing model (Fig. 1c)^28^. Mice that express Cre recombinase under control of the endogenous *Foxa2* promoter through use of the autocleaving 2A linker, *Foxa2*^*T2AiCre*^ (*Foxa2Cre*)^28^, were crossed to *Rosa26-tdTomato* (*tdT*) reporter mice^29^,^30^, to generate *Foxa2Cre:tdT* lineage-tracing mice in which endogenous *Foxa2* expression remains undisturbed. In *Foxa2Cre:tdT* mice, cells that have expressed *Foxa2* at any time during their development will remain tdT positive, irrespective of endogenous *Foxa2* expression at later stages.

When *Foxa2Cre:tdT* embryos are collected during late gastrulation (E7.5), tdT+ cells are found predominantly in the node, midline, and visceral and definitive endoderm, as expected, as well as in a region that corresponds to migrating mesoderm cells (Fig. 1d, arrowhead). Interestingly, tdT+ cells can further be observed on the anterior side of the embryo in the CC region at E8.25 (Fig. 1e, arrowhead). At E8.5, when the PHT begins to form, tdT+ cells are present in the ventral region of the PHT, indicating contribution of Foxa2-expressing cells to the forming heart and distinct localization of these cells early during cardiac morphogenesis (Fig. 1f, arrowhead). As the heart continues to develop into a regionalized structure with four distinct chambers (E9.5–17.5), tdT+ cells become localized to the ventricular chambers, a pattern that persists throughout development (Fig. 1g–j). Identical lineage-tracing patterns are observed with a different reporter allele (*Rosa26-YFP*)^31^ crossed to the *Foxa2Cre* line (Supplementary Fig. 1). Live *en face* images, as well as sections and immunofluorescence (IF) analysis of the corresponding time points further illustrate the distinct distribution of *Foxa2Cre:tdT* linage-traced cells (Supplementary Figs 2 and 3a,b). Importantly, endogenous Foxa2 expression is not detected in any cardiac structures from E8.5 onwards (Supplementary Fig. 3a–c), confirming that Foxa2 is not expressed in cardiomyocytes, and suggesting that *Foxa2* lineage-traced, ventricular cells (tdT+) are derived from a population preceding the differentiated heart.

As Foxa2 lineage-traced cells present in the PHT no longer express Foxa2 (Supplementary Fig. 3a–c), we next sought to determine the precise time of occurrence of the Foxa2+ cardiac progenitor population during development. To this end, we performed inducible lineage tracing by crossing mice carrying a tamoxifen (Tam)-inducible Cre recombinase expressed under the control of the endogenous *Foxa2* promoter^32^ with *Rosa26-tdT* mice (*Foxa2CreER:tdT*). We injected Tam at distinct time points during development (E6.5–E8.5) and embryos were analysed at E12.5 to assess the distribution of tdT+ cells in the heart. Tam injection at E6.5 results in the presence of tdT+ cells in both ventricular chambers but not the atria of the heart, consistent with the pattern observed in the constitutive *Foxa2* lineage-tracing embryos (Fig. 1k). However, embryos injected with Tam at E7.5 or later were devoid of tdT+ cells in the heart (Fig. 1l,m), supporting the hypothesis that *Foxa2* expression in cardiac progenitor cells is transient and occurs during gastrulation. As expected, the labelling efficiency using inducible lineage tracing is lower compared with lineage tracing with the constitutively active *Foxa2Cre* line, likely due to the low doses of Tam (0.05 mg g^−1^) that are required to avoid toxicity to the developing embryos. Consistent with the maintained expression of *Foxa2* in the liver and lung throughout development, tdT+ cells can readily be observed in these organs when Tam is injected at the later time points (Supplementary Fig. 4). Importantly, these data confirm that *Foxa2*-expressing progenitor cells with ventricular differentiation potential, hereafter referred to as Foxa2-vCPs, are specified during gastrulation, and that later expression of Foxa2 is excluded from the differentiated cardiovascular lineages. Collectively, our data describe a unique progenitor cell population during gastrulation that is marked by the expression of a single gene, *Foxa2*, and that distinctly segregates into atrial–ventricular chambers later in development.

### Foxa2-vCPs contribute to cardiac mesoderm

**Figure 2:**
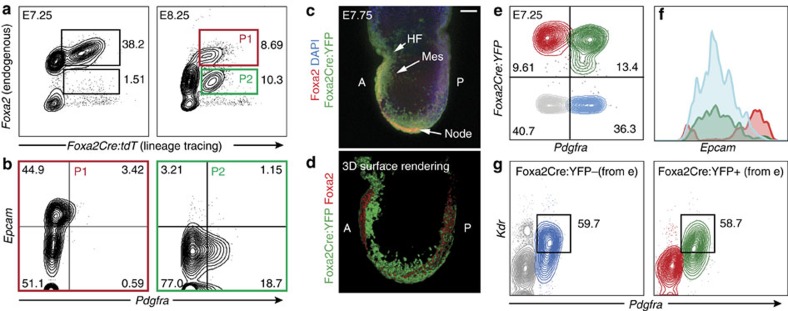
*Foxa2* expression marks a transient progenitor population during gastrulation that gives rise to CM. (**a**) Flow cytometry analysis of dissociated E7.25 (left) or E8.25 (right) *Foxa2Cre:tdT* embryos. Cells were stained with antibodies against endogenous Foxa2, which was compared with the *Foxa2Cre:tdT* lineage tracing marker. (**b**) Foxa2+tdT+(P1, left panel) and Foxa2-tdT+(P2, right panel) cells from E8.25 embryos were further analysed with antibodies against Epcam (endoderm) and Pdgfra (mesoderm). (**c**) Confocal *z*-projection of E7.75 *Foxa2Cre:YFP* embryo analysed by WMIF using antibodies against YFP and Foxa2. Scale bar, 75 μm. (**d**) Three-dimensional (3D) surface rendering of image shown in **c** generated using Imaris software. (**e**–**g**) Flow cytometry analysis of dissociated E7.25 *Foxa2Cre:YFP* embryos with antibodies against Pdgfra, Epcam and Kdr. Cells were first gated based on expression of YFP and Pdgfra (**e**). The resulting quadrants were then plotted on a histogram for Epcam (**f**) to assess for endodermal identity of the cells. In addition, the YFP− (grey/blue) and YFP+ (green/red) cells were separately analysed for expression of the CM markers Pdgfra and Kdr (**g**). A, anterior; P, posterior; HF, head folds; Mes, mesoderm.

To confirm the transient nature of *Foxa2* expression in Foxa2-vCPs, we analysed *Foxa2Cre:tdT* lineage-traced embryos at different stages of development for endogenous Foxa2 protein expression via flow cytometry. As expected, all tdT+ cells also express endogenous Foxa2 during gastrulation (Fig. 2a, E7.25). At slightly later stages, in addition to the Foxa2+tdT+ cells (Fig. 2a, E8.25, P1), a subpopulation of tdT+ cells emerges that no longer expresses endogenous Foxa2 protein (Fig. 2a, E8.25, P2). When investigating the expression of the mesoderm marker Pdgfra and the endoderm marker Epcam^33^, many of the Foxa2+tdT+ (P1) cells co-express Epcam, suggesting partial endoderm identity of this population. However, Foxa2-tdT+ (P2) cells do not express Epcam but contain a subpopulation of cells expressing Pdgfra, indicating a mesodermal identity (Fig. 2b). Transient Foxa2 expression was further confirmed by whole-mount IF (WMIF) of *Foxa2Cre:YFP* embryos for yellow fluorescent protein (YFP) and Foxa2 at E7.75, which again illustrates the presence of both Foxa2+YFP+ and Foxa2-YFP+ cell populations (Fig. 2c). To facilitate visualization of these populations, surface renderings were generated based on the confocal images in Fig. 2c. These renderings clearly show that endogenous Foxa2 is restricted to the midline of the developing embryo, whereas the YFP+ region extends laterally from the anterior side (Fig. 2d). To further investigate the mesoderm identity of the Foxa2-derived cells, we crossed *Foxa2Cre:tdT* mice to *Pdgfra-H2B-GFP* mice^34^ (Supplementary Fig. 5a,b). WMIF analysis of E7.5 *Foxa2Cre:tdT/Pdgfra-H2B-GFP* embryos with antibodies for endogenous Foxa2 revealed Foxa2+tdT+ (Foxa2-expressing endoderm and ectoderm lineages) and GFP+tdT− (Pdgfra-expressing mesoderm) populations, as expected, and that endogenous Foxa2 and Pdgfra expression are largely exclusive (Supplementary Fig. 5c). Interestingly, we do observe GFP+tdT+ cells, primarily located at the anterior side of the embryo, where the CC is forming at that stage during development (Supplementary Fig. 5c).

The mesoderm cells that will develop into the cardiac structures are formed shortly after gastrulation and are well known to co-express Pdgfra and Kdr (Flk-1)^35^,^36^,^37^,^38^. To assess whether a subpopulation of CM is derived from Foxa2-vCP cells we performed flow cytometry analysis on E7.25 embryos for YFP (*Foxa2Cre:YFP*), Epcam (endoderm) and Pdgfra/Kdr (CM) (Fig. 2e–g). Interestingly, we detect equal populations of Kdr-expressing cells in Pdgfra+YFP+ and Pdgfra+YFP− populations, indicating that Foxa2-vCPs do indeed contribute to and develop through a CM stage (Fig. 2g). Furthermore, compared with YFP+Pdgfra− cells (red population), YFP+Pdgfra+ (green population) cells do not express Epcam, again illustrating that they are not of endodermal identity (Fig. 2f). Taken together, our data describe a cell population that transiently expresses Foxa2 during early development and demonstrate that Foxa2-derived cells give rise to a subset of CM in the embryo.

### *In vitro* characterization of Foxa2-derived cardiac progenitors

**Figure 3:**
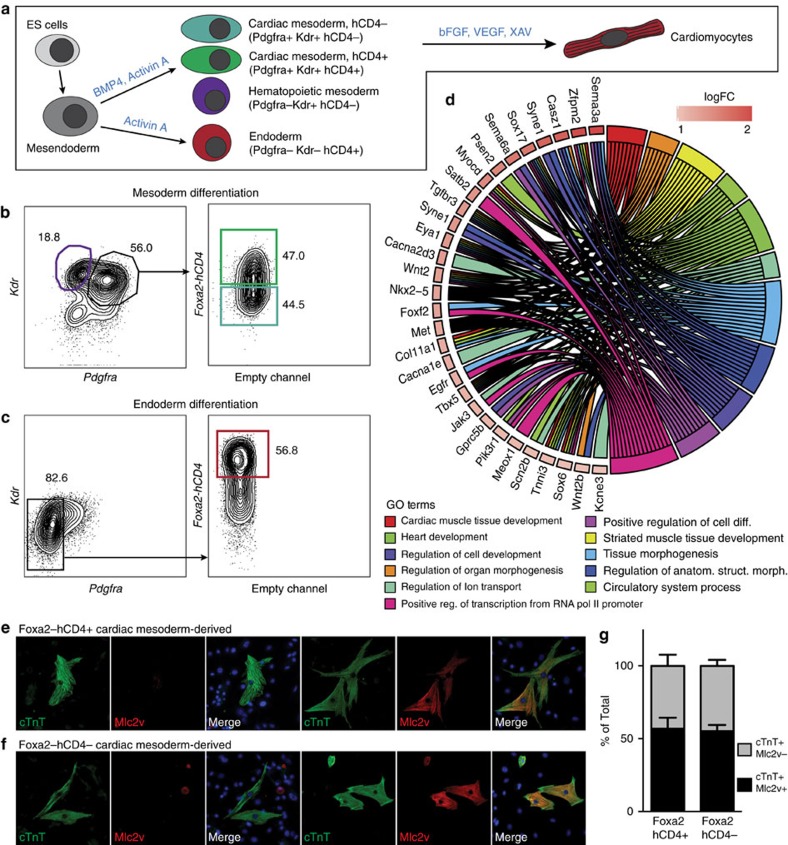
Foxa2+ CM cells can be identified and characterized during mESC differentiation. (**a**) Schematic of mESC *in vitro* differentiation protocols to the cardiovascular and endoderm lineages. Mesoderm cells are generated through addition of Bmp4 and Activin A, whereas endoderm cells are generated through addition of high levels of Activin A. CM is specified to the cardiomyocyte lineage through addition of basic fibroblast growth factor (bFGF), vascular endothelial growth factor (VEGF) and XAV. (**b**,**c**) Fluorescence-activated cell sorting (FACS) strategies for the isolation of Foxa2+ and Foxa2− CM (green and teal gates, respectively), Kdr^high^ haematopoietic mesoderm (purple gate) or Foxa2+ endoderm (red gate) at day 5 of differentiation. (**d**) Chord plot showing a selection of genes upregulated in Foxa2+ CM (over Foxa2− CM) present in the represented enriched GO terms. Outer ring shows log2 fold change (left, key at upper right) or GO term grouping (right, key below). Chords connect gene names with GO term groups. (**e**,**f**) IF analysis of differentiated cardiomyocytes generated from Foxa2+ (**e**) or Foxa2− (**f**) CM showing expression of cTnT and Mlc2v. Images show typical cells generated in each condition. (**g**) Quantification of experiments in (**e**/**f**) (*n*=3 differentiations, error bars reflect s.e.m.).

Regenerative approaches and differentiation strategies using PSCs have strongly benefitted from translating developmental concepts to *in vitro* culture systems^39^,^40^. To investigate whether Foxa2-vCPs can be generated *in vitro*, we assessed *Foxa2* expression during the differentiation of mESCs to cardiomyocytes. To monitor *Foxa2* expression we used a mESC line that expresses a truncated human CD4 from the *Foxa2* locus (*Foxa2-hCD4*)^41^. *Foxa2-hCD4* mESCs were differentiated using a cardiac specific differentiation protocol (Fig. 3a)^36^ and the emergence of CM was assessed by flow cytometry analysis for expression of Kdr and Pdgfra at day 5 of differentiation (Fig. 3b and Supplementary Fig. 6). As expected, based on the analysis of *Foxa2*Cre:YFP mouse embryos, a large portion of the Kdr+Pdgfra+ CM cells also expresses hCD4 (green gate). As a control, *Foxa2-hCD4* mESCs were differentiated using an endoderm differentiation protocol (Fig. 3a)^42^, which results in cultures containing very high Foxa2-expressing cells (red gate), which are negative for the mesoderm markers Pdgfra and Kdr (Fig. 3c).

To characterize Foxa2+ and Foxa2− CM for potential atrial–ventricular specification mechanisms, we isolated hCD4+ and hCD4− CM, as well as Foxa2-hCD4+ endoderm and Kdr+ haematopoietic mesoderm (Fig. 3b, purple gate) by fluorescent activated cell sorting and performed RNA sequencing (RNAseq) analysis. Principal component analysis and calculation of the Euclidian distance between the rlog-transformed data for each sample demonstrates the strong similarity of Foxa2+ CM and Foxa2− CM samples, and their collective difference from either endoderm or haematopoietic mesoderm (Supplementary Fig. 7a,b). This analysis further illustrates that the mesoderm populations are more closely related to each other than to Foxa2+ endoderm cells, confirming again the existence of a CM population derived from a Foxa2-expressing progenitor during development. Scatterplots comparing rlog-transformed read counts between representative replicates demonstrate global differences in gene expression, supporting the relationships observed through clustering (Supplementary Fig. 7c).

Differential expression analysis performed using DESeq2 reveals 119 genes more than 2-fold differentially expressed between Foxa2+ CM and Foxa2− CM (Supplementary Figs 7d and 8a), and 620 genes at least 1.5-fold differentially expressed. Gene Ontology biological process enrichment analysis for genes upregulated at least 1.5-fold in Foxa2+ CM reveals a number of significantly enriched pathways, including those related to heart development and organ morphogenesis (Fig. 3d). Included in this list are *Sema3a*, which is known to play a key role in controlling heart rhythm^43^; *Casz1*, a transcription factor important for proper heart morphogenesis and tissue integrity^44^,^45^; and *Zfpm2* (or *Fog2*), a GATA-interacting protein that is highly implicated in congenital heart defects^46^,^47^. Gene expression for a selection of candidates from these pathways including *Myocd*, *Zfpm2*, *Sema3a*, *Tbx5*, *Alox15*, *Syne1*, *Sema6a*, *Isl1* and *Mesp1* was evaluated by quantitative reverse transcriptase–PCR to confirm the results concluded by DESeq2 (Supplementary Fig. 8b). In addition, WMIF of E7.5 *Foxa2Cre:YFP* embryos revealed that Myocd and Zfpm2 protein can be detected within the *Foxa2Cre:YFP+* cell population (Supplementary Fig. 8c,d).

To assess the cardiac differentiation potential of Foxa2+ CM cells, we next isolated Foxa2− CM and Foxa2+CM cells from mESC cultures and differentiated the cells towards the cardiomyocyte lineage. IF analysis for cardiac Troponin T (cTnT) and Mlc2v after 5 weeks of differentiation indicates that both populations are able to give rise to cardiomyocytes, and that a subset of these cardiomyocytes express Mlc2v, indicating ventricular identity (Fig. 3e,f). Quantification of the percentage of ventricular cardiomyocytes generated from Foxa2+ or Foxa2− CM reveals similar proportions, suggesting a plasticity of the cell culture system and/or the maintained ventricular potential of Foxa2− CM. Collectively, this analysis confirms that Foxa2-hCD4+ CM cells can be generated *in vitro*, and that they express genes important for heart development and function, and furthermore reveals genes that may be relevant during the earliest steps of ventricular specification.

### Foxa2-vCPs contribute to the first and second heart fields

**Figure 4:**
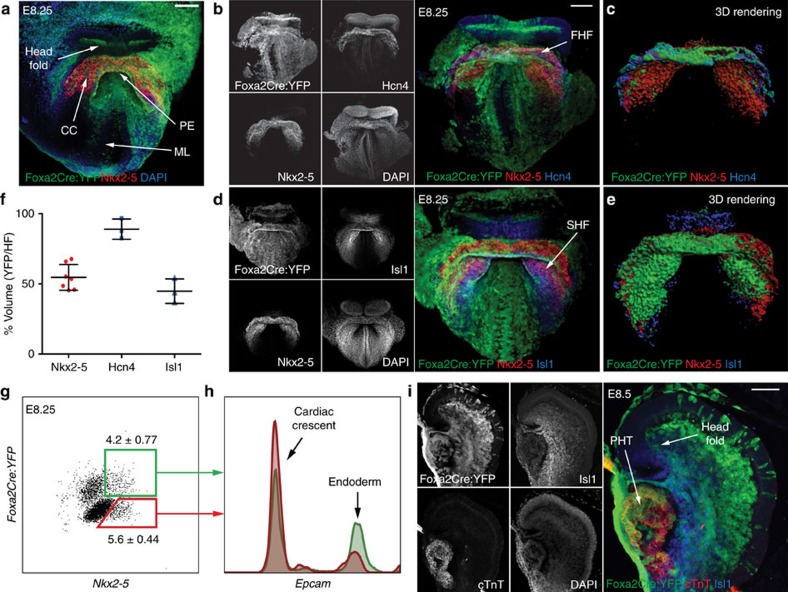
Foxa2-vCPs contribute to the FHF and SHF during heart morphogenesis. (**a**) Confocal *z*-projection of E8.25 *Foxa2Cre:YFP* embryos analysed by WMIF using antibodies against YFP and Nkx2–5. (**b**–**e**) WMIF analysis of E8.25 *Foxa2Cre:YFP* embryos with antibodies against Hcn4 (**b**) or Isl1 (**d**), and three-dimensional (3D) surface renderings of the CC region as generated with Imaris software (**c**,**e**). Representative stage-matched embryos are shown. (**f**) Quantification of the volume of the 3D surface rendering showing the YFP volume as a percentage of the total CC (Nkx2–5), FHF (Hcn4) or SHF (Isl1). Error bars reflect s.d. (**g**) Flow cytometric analysis of dissociated E8.25 *Foxa2Cre:YFP* embryos with antibodies against Nkx2–5. (**h**) Analysis of Nkx2–5+YFP+ and Nkx2–5+YFP− cell based on Epcam expression. (**i**) WMIF analysis of E8.5 *Foxa2Cre:YFP* embryo with antibodies against Isl1 and cTnT. CC, cardiac crescent; FHF, first heart field; ML, midline; PE, pharyngeal endoderm; PHT, primitive heart tube; SHF, second heart field. Scale bars, 100 μm.

We next sought to elucidate how the Foxa2-vCP lineage aligns with our current understanding of cardiac progenitor morphogenesis and differentiation within the CC, which is the stage immediately before formation of the PHT. The CC is defined by Nkx2–5 expression, and WMIF of E8.25 *Foxa2Cre:YFP* embryos shows that Foxa2-vCPs contribute to a large population of cells within the CC (Fig. 4a). Interestingly, YFP+ cells localize to a distinct domain within the Nkx2–5+ cell population, which is predominantly located at the apex of the CC (Fig. 4a).

To visualize the localization of Foxa2-vCP derivatives within the FHF and SHF regions of the CC, we performed additional analysis with markers for these specific progenitor populations. Staining with Hcn4 labels a specific region within the Nkx2–5 domain corresponding to the FHF. Consistent with the contribution of Foxa2-vCPs to the FHF-derived left ventricle, YFP+ cells are found abundantly within the Hcn4+ region (Fig. 4b and Supplementary Movies 1 and 2). WMIF for Islet 1 (Isl1) shows labelling throughout the SHF region of the CC, as expected. YFP+ cells are found at the anterior-most region of the SHF, again consistent with the contribution of Foxa2-vCPs to the SHF-derived RV (Fig. 4d, Supplementary Fig. 9 and Supplementary Movies 3–5).

To quantitatively determine the contribution of Foxa2-vCPs to the FHF and SHF regions, we extended the analysis of the CC regions and performed surface rendering of the confocal images. Specifically, using Imaris software, surfaces were generated for the reference stains Nkx2–5 (CC), Hcn4 (FHF) and Isl1 (SHF). These surfaces were then used to mask the YFP+ signal in order to select only the YFP+ regions that fall within each of the areas of interest. A surface rendering was then generated for the masked YFP signal (Fig. 4c,e and Supplementary Movies 6 and 7, note that presented colours do not indicate channel merges). The volume of the YFP surface was calculated and compared with the volume of each of the reference surfaces and is displayed as a percentage of total volume (Fig. 4f). Based on this analysis we conclude that ∼50% of the total CC (Nkx2–5+) is derived from Foxa2-vCPs, consistent with the contribution of Foxa2-vCPs at the CM stage (Fig. 2g). Based on the same analysis we conclude that Foxa2-vCPs give rise to a majority of the FHF and about half of the SHF (Fig. 4f); this is consistent with the ventricular differentiation potential of the Foxa2-vCP population, as the FHF gives rise primarily to the LV, while the SHF gives rise to the RV in addition to other regions of the heart^2^,^9^,^10^. To further support our image-based volumetric analysis, we additionally performed flow cytometric analysis for Foxa2-vCP-derived cells and the CC regions of individual E8.25 embryos using antibodies against Nkx2–5 and Epcam (to exclude the pharyngeal endoderm). This analysis illustrates highly similar numbers of YFP+ (43%±7.7% of total Nkx2–5, *n*=8) and YFP− (57%±4.4% of total Nkx2–5, *n*=8) cells in the CC overall (Fig. 4g,h), thus confirming the results generated using the three-dimensional surface rendering analysis.

We next sought to relate the Foxa2-vCP-derived population to the recently described Hopx+ cardiomyoblast progenitor cells^48^. WMIF analysis of Hopx-GFP embryos with antibodies against Isl1 and Nkx2–5 reveals few Hopx-GFP+ cells within the CC, as previously reported^48^ (Supplementary Fig. 10a), and confirms the localization of Hopx-GFP+ cells within the transition zone between Isl1+ cells and the heart tube at later stages (Supplementary Fig. 10b,c). This suggests that Foxa2-vCPs migrate to and organize within the CC before Hopx expression and execution of the differentiation programme.

Following the formation of the CC, cardiac precursors undergo differentiation and begin growing outward to produce the PHT. It is during the heart tube growth and looping processes that chamber identity and morphology are established. With this in mind, we used WMIF to determine the localization of the Foxa2-vCP-derived cells within the heart tube, labelled with cTnT, in E8.5 *Foxa2Cre:YFP* embryos (Fig. 4i and Supplementary Movies 8 and 9). As expected, YFP+ cells in these embryos are found within the ventral region of the heart tube, which will give rise to ventricular structures.

In summary, the data shown here illustrate that Foxa2-vCPs give rise to subsets of the FHF and SHF structures at the CC stage of heart development and subsequently to the ventral region of the growing heart tube. These observations thus suggest that prospective ventricular structures of the heart can be distinguished by *Foxa2* lineage tracing as early as during gastrulation and throughout heart development.

### Foxa2-vCPs contribute to all ventricular cardiovascular lineages

**Figure 5:**
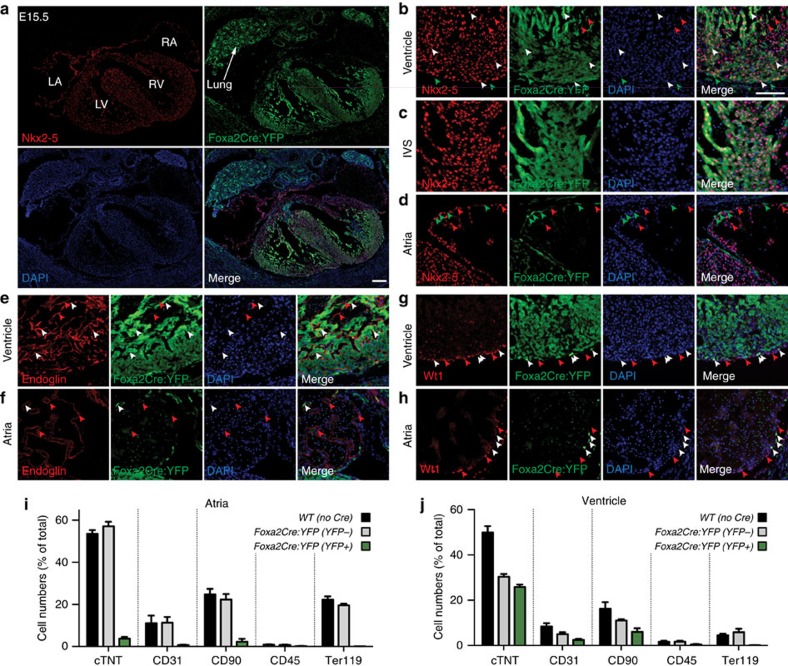
Contribution of Foxa2-vCPs to the major cardiovascular lineages. (**a**) Heart sections from E15.5 *Foxa2Cre:YFP* embryos stained with antibodies against YFP and Nkx2–5 to label cardiomyocytes. Tile scan image showing heart and lung. (**b**–**h**) IF analysis of E15.5 *Foxa2Cre:YFP* embryos using antibodies against YFP and Nkx2–5 (myocardium, **b**–**d**), Endoglin (endocardium, **e**,**f**) or Wt1 (epicardium, **g**,**h**). Detailed areas of ventricle (**b**,**e**,**g**), interventricular septum (**c**) and atria (**d**,**f**,**h**) are shown. Arrowheads indicate cells that express the relevant lineage markers alone (red) or that co-express YFP and the relevant lineage marker (white). Images are representative examples from multiple experiments. (**i**,**j**) Flow cytometry analysis of dissociated cells of atrial (**i**) and ventricular (**j**) chambers of E13.5 *Foxa2Cre:YFP* hearts with antibodies against cTnT (cardiomyocytes), CD31 (endothelial), CD90 (mesenchymal, haematopoietic, fibroblast, epicardium), CD45 (leukocyte) and Ter119 (erythroid). Data are mean±s.e.m. of *n*=7 hearts. Scale bars, 100 μm. LA, left atria; LV, left ventricle; RA, right atria; RV, right ventricle.

**Figure 6:**
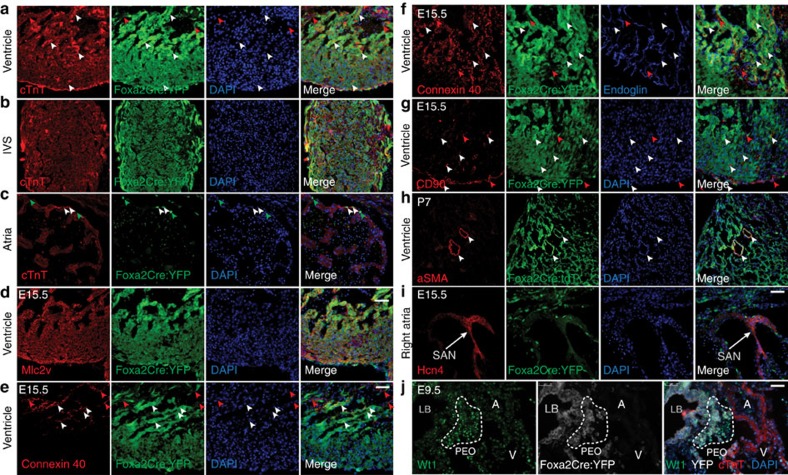
Contribution of Foxa2-vCPs cells to the differentiated lineages in the heart. (**a**–**i**) IF analysis of E15.5 embryos (**a**–**g**,**i**) or P7 hearts (**h**) using antibodies against YFP or tdT and cTnT (cardiomyocytes, **a**–**c**) Mlc2v (ventricular-specific ion channel, **d**), Connexin 40 (conduction system, **e**), Connexin 40 and Endoglin (conduction and endothelial cells, **f**), CD90 (fibroblasts and epicardium, **g**), aSMA (smooth muscle, **h**) or Hcn4 (SA node, **i**). (**j**) IF analysis of E9.5 embryos with antibodies against Wt1 (PEO). Arrowheads indicate cells that express the relevant markers (red) or that co-express YFP and the relevant marker (white). Dashed line indicates the area of the PEO at E9.5. All images shown are representative of multiple experiments. A, atria; LB, liver bud; PEO, proepicardial organ; V, ventricle. Scale bars, 50 μm.

The mature heart is composed of multiple cell types, including myocardium, endocardium, epicardium, smooth muscle cells and conduction system cells. To characterize the lineages that arise from Foxa2-vCPs during development, we performed IF analysis for markers of the cardiovascular lineages on *Foxa2Cre:YFP* embryos at E15.5. This analysis demonstrates that Foxa2-vCPs give rise to cardiomyocytes (Nkx2–5, cTnT and MLC2v), endothelial/endocardial cells (endoglin), epicardium (Wilm's tumour 1, Wt1), fibroblasts (CD90), smooth muscle (aSMA), and conduction system cells (Cx40) in both ventricles and in the interventricular septum (Figs 5a–c,e,g and 6a,b,d–h). In accordance with our observations in the constitutive lineage-tracing model, induction of Tam-inducible *Foxa2CreER* at E6.5 results in labelling of Foxa2-vCPs that contribute to the myocardium (cTnT), endocardium (endoglin) and epicardium (Wt1) of the developed ventricles (Supplementary Fig. 12). Consistent with the lineage tracing above, only very few cells of the myocardial, endothelial or sinoatrial node in the atria expressed YFP, indicating that they are primarily not derived from Foxa2-vCPs (Figs 5d,f and Supplementary Fig. 11a–d).

The primary Foxa2-vCP-derived cells in the atria consist of a subpopulation of epicardial cells expressing Wt1 (Fig. 5h). The epicardium develops separately from the heart tube structure as it is derived from the PEO, which is located posteriorly to the developing heart tube at E9.5. From there, PEO cells migrate out to ultimately encase the entire heart. As predicted by the YFP+Wt1+ epicardial cells in the differentiated heart, we found that cells of the PEO co-expressed YFP and Wt1, indicating that Foxa2-vCPs colonize this tissue early in development and therefore give rise to epicardial cells of the entire heart (Fig. 6j).

The cardiovascular lineage analysis further illustrates that not all ventricular cells are YFP+. To assess this in more detail, we quantified the allocation of YFP+ cells to multiple lineages in the differentiated heart by flow cytometry. As predicted, minimal overlap was observed between YFP and markers for cardiomyocytes (cTnT), endothelial (CD31), leukocytes (CD45), mesenchymal/haematopoietic (CD90) or erythroid (Ter119) cells in the atria, indicating that these lineages are largely not derived from Foxa2-vCPs (Fig. 5i and Supplementary Fig. 13a). In contrast, YFP+ ventricular cells co-stain with cells expressing cTnT, CD31 and CD90, but not with markers specific to the haematopoietic lineage (Fig. 5j and Supplementary Fig. 13b). Additional flow cytometry quantification of the Foxa2 lineage-traced cardiomyocytes in the left versus right ventricular chambers illustrates that Foxa2-vCPs contribute to both chambers in similar amounts (Supplementary Fig. 14).

Taken together, these data demonstrate that Foxa2-vCPs give rise to diverse cardiovascular lineages of both ventricular chambers of the differentiated heart, as well as to the epicardium of all chambers. Despite the long-standing hypothesis that specification events relevant to the heart occur early during development, this is the first time that cells with predominantly ventricular differentiation potential can be genetically labelled and thus followed over time, via expression and subsequent lineage tracing of *Foxa2*. As such, our findings offer a new approach for further dissecting cardiovascular lineage specification events during early heart development.

### Foxa2 is required for the formation of ventricular cells

**Figure 7:**
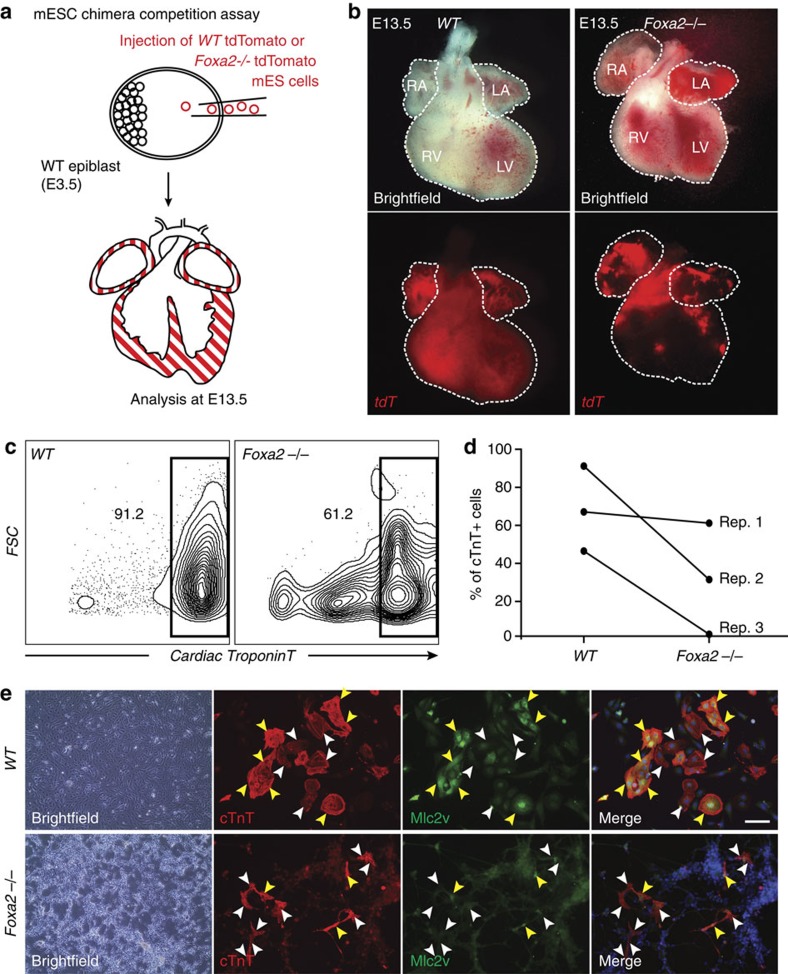
Foxa2 is necessary for the generation of ventricular cells during cardiac development. (**a**) Schematic of mESC chimera competition assay. Fluorescently labelled *WT* or *Foxa2−/−* mESCs are injected into unlabelled *WT* blastocysts at E3.5. Embryos are collected at E13.5 and the distribution of tdT+ cells is observed. (**b**) Whole-mount imaging of typical *WT* (left) and *Foxa2−/−* (right) mESC-injected hearts at E13.5. (**c**) Flow cytometry analysis of cell populations differentiated from *WT* and *Foxa2−/−* mESC cells. Cells at day 10 of differentiation were dissociated and analysed by flow cytometry for the cardiac marker cTnT. (**d**) Quantification of **c**. Paired data are plotted for *n*=3 replicates. (**e**) Cells at day 10 of differentiation from *WT* and *Foxa2−/−* mESCs were plated and IF analysis was performed with antibodies against cTnT (red) and the ventricular-specific marker Mlc2v (green). Yellow arrowheads illustrate overlap of cTnT and Mlc2v. White arrowheads indicate cTnT cells that are not stained for Mlc2v. Scale bar, 50 μm. LA, left atria; LV, left ventricle; RA, right atria; RV, right ventricle.

Following the identification of a Foxa2+ progenitor population with ventricular-specific differentiation potential, we next sought to determine the role of Foxa2 during heart development. As complete loss of Foxa2 results in embryonic lethality due to severe defects in endodermal and ectodermal lineages^22^, the Foxa2-null mouse model is not suitable for detailed studies of potential cardiac phenotypes. As an alternative approach to assess whether Foxa2-deficient cells can contribute to the heart, we performed mESC mixed chimera competition assays using mES cells labelled with Lyn-tdT^49^. Wild-type (WT) or Foxa2-null mESCs (*Foxa2−/−*) were injected into unlabelled WT E3.5 blastocysts and the chimeric embryos were collected at E13.5 (Fig. 7a). In this mosaic analysis, WT cells contribute evenly to all regions of the chimeric embryos (Supplementary Fig. 15a). As expected, tissues that rely strongly on Foxa2 for their development, such as the liver and notochord, are completely devoid of *Foxa2−/−* cells (Supplementary Fig. 15a). After dissection of the heart, we monitored distribution of tdT+ cells to determine the ability of *WT* and *Foxa2−/−* cells to contribute to the different chambers of the heart. Interestingly, the majority of embryos injected with *Foxa2−/−* mESCs (75%, 9/12) displays a complete lack of tdT+ cells in the apex of the ventricles, in a pattern reminiscent of the *Foxa2Cre:YFP* lineage-tracing results, whereas the atria of these hearts do contain tdT+ cells (Fig. 7b). Hearts that contain some *Foxa2−/−* tdT+ cells in the ventricles (25%, 3/12) show abnormal ventricular morphology (Supplementary Fig. 15b). Size measurements further revealed that the resulting ventricular chambers are significantly smaller compared to either *WT* or *Foxa2−/−* tdT− ventricles (Supplementary Fig. 15c). These data suggest a cell-autonomous function for Foxa2 during development and morphogenesis of the heart.

To support these findings, we next investigated whether *Foxa2−/−* mESCs can differentiate to the cardiovascular lineage *in vitro*. *Foxa2−/−* mESCs are able to form embryoid bodies (EBs) normally as expected; they are also able to differentiate to cardiomyocytes, but the efficiency, as measured by generation of cTnT+ cells, is consistently reduced compared with *WT* ESCs (Fig. 7c,d). When EBs are dissociated, plated and stained for the ventricular myocyte marker Mlc2v, we observe a decrease in Mlc2v+cTnT+ cardiomyocytes in *Foxa2−/−* compared with *WT* cultures (Fig. 7e), indicating that cardiomyocytes derived from Foxa2−*/*− mESCs predominantly display an atrial phenotype during differentiation. This is in strong correlation with our findings in the *in vivo* mESC chimera experiments and suggests that Foxa2 does not solely mark a cell population fated to give rise to ventricular cardiomyocytes, but raises the possibility that it is required for the correct specification and development of the ventricular chambers of the heart.

### Foxa2-vCP-specific ablation of Isl1 perturbs morphogenesis

**Figure 8:**
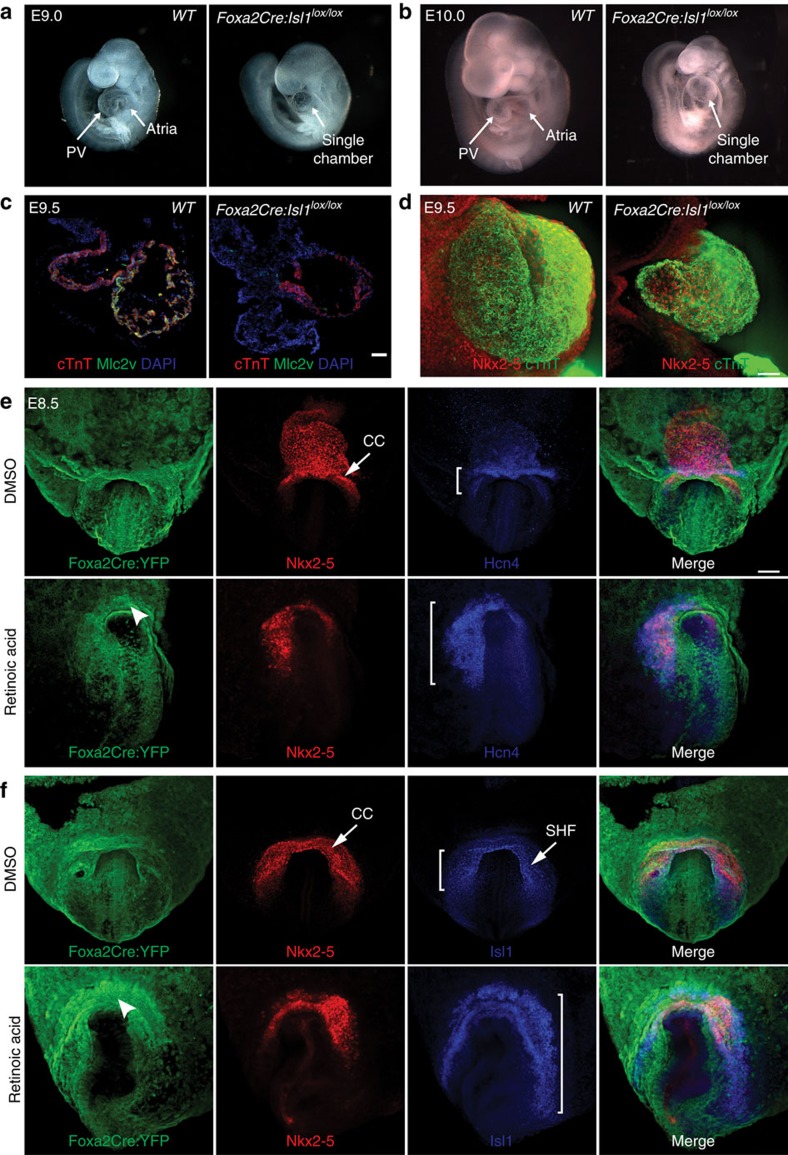
Perturbation of the Foxa2-vCP population results in aberrant cardiac morphology. (**a**,**b**) *WT* (left) and *Foxa2Cre:Isl1*^*lox/lox*^ (right) embryos were imaged for morphological differences at E9.0 (**a**) and E10.0 (**b**). (**c**) IF analysis of cryosectioned E9.5 *WT* and *Foxa2Cre:Isl1*^*lox/lox*^ embryos with antibodies against cTnT and Mlc2v. (**d**) WMIF and confocal *z*-projection of E9.5 *WT* and *Foxa2Cre:Isl1*^*lox/lox*^ embryos with antibodies against Nkx2–5 and cTnT to label the growing heart tube. (**e**,**f**) WMIF analysis of E8.5 *Foxa2Cre:YFP* embryos injected at E7.5 with either dimethylsulfoxide (DMSO; vehicle control, top rows) or RA (65 mg kg^−1^, bottom rows) and labelled with antibodies against YFP, Nkx2–5 and either Hcn4 (**e**) or Isl1 (**f**). Brackets indicate expansion of heart field region after RA treatment. Images shown are representative (*n*=3 embryos for each stain). PV, primitive ventricle; CC, cardiac crescent; SHF, second heart field. Scale bars, 75 μm (**c**) or 100 μm (**d**,**e**).

To further probe the relevance of Foxa2-vCPs for the development of the heart, we investigated whether Isl1, a key regulator of cardiac differentiation expressed early during heart development, is required in Foxa2-vCPs. To this end, *Foxa2Cre* mice were crossed to Isl1^lox/lox^ mice^50^, to generate embryos deficient for Isl1 in Foxa2-vCPs (*Foxa2Cre:Isl1*^*lox/lox*^) and their derivatives. *Foxa2Cre:Isl1*^*lox/lox*^ embryos do not survive past mid-gestation due to defects in multiple structures, including the heart. At E9.0 and E10.0, we observe contraction in hearts of *Foxa2Cre:Isl1*^*lox/lox*^ embryos; however, these hearts do not loop into distinct chambers but rather resemble the PHT (Fig. 8a,b), similar to what had been observed in Isl1-null embryos^51^. IF analysis for cTnT and the ventricular marker Mlc2v confirms the reduced size of *Foxa2Cre:Isl1*^*lox/lox*^ hearts and illustrate a decrease in cTnT+Mlc2v+ cardiomyocytes of the heart tube (Fig. 8c). To further characterize the looping defect observed in these embryos, we performed WMIF of E9.5 *WT* and *Foxa2Cre:Isl1*^*lox/lox*^ embryos. Heart tubes of *WT* embryos show the distinct looped tube morphology typical for this stage of development (Fig. 8d and Supplementary Movies 10 and 11). In *Foxa2Cre:Isl1*^*lox/lox*^ embryos, however, the heart tube extends outward ventrally from the body, forming a large single cavity (Fig. 8d and Supplementary Movies 12 and 13). These findings show that perturbations to the canonical cardiac gene regulatory network within the Foxa2-vCPs result in erroneous development and aberrant morphology, and that non-Foxa2-vCPs are unable to compensate for these deficiencies.

### RA signalling induction hinders Foxa2-vCP differentiation

The role of retinoic acid (RA) in heart development has been widely described in multiple model systems^52^. Several of these studies have shown that treatment with exogenous RA promotes atrial identity within the cardiac field^53^,^54^. To determine how exposure to RA might affect the differentiation of Foxa2-vCPs, we exposed E7.5 embryos to a pulse of exogenous all-*trans* RA (RA injection of pregnant females). Embryos were dissected and analysed by WMIF at E8.5 for YFP and expression of markers for the cardiac crescent (Nkx2–5), FHF (Hcn4, marks sinus venosus progenitors in later stages) and SHF (Isl1) (Fig. 8e,f). Embryos treated with RA at E7.5 exhibit mild developmental delays compared with dimethylsulfoxide-treated controls. Interestingly, after RA treatment the Isl1+ region extends posteriorly (Fig. 8f), a phenotype that has previously been seen after deletion of *Raldh2* (refs 55, 56). With respect to the Foxa2-vCP population, YFP+ cells populate the anterior region of the CC, indicating that their specification between E6.5–7.5 is unperturbed. Nevertheless, the anterior-most cells show low expression of Nkx2–5, suggesting an inability to properly execute the cardiac differentiation programme (Fig. 8e,f). Based on these data, it is therefore conceivable that the increased acquisition of atrial identity after RA treatment is not due to an inability to specify ventricular progenitors, but rather stems from an inability of ventricular progenitors to fully differentiate and contribute to the growing heart tube.

## Discussion

Clonal analysis has suggested that regional segregation and lineage restriction of cardiac progenitors occurs early during gastrulation^20^,^21^, which is in accordance with fate maps generated in the fish, chick and mouse embryos^13^,^16^,^19^,^25^. This segregation appears to occur largely along the FHF and SHF lineages, and thus atrial–ventricular boundaries have remained elusive to date. Through genetic lineage tracing we have identified a progenitor population that specifically develops into the cardiovascular cells of both ventricles of the differentiated heart but not the atria. Importantly, this ventricular-specific progenitor population emerges during gastrulation and our data thus strongly support previous hypotheses that atrial–ventricular segregation occurs long before the establishment of differentiated cardiac structures.

**Figure 9:**
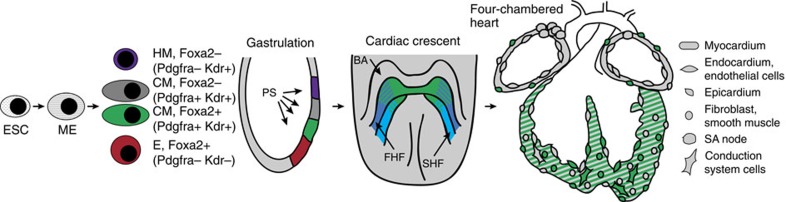
Schematic illustrating the emergence and subsequent pattern of contribution of Foxa2-vCPs during cardiac development. Foxa2-vCPs (indicated in green) emerge during gastrulation (E6.5–7.5). Progeny derived from the Foxa2-vCPs (continued in green) start expressing the CM markers Pdgfra and Kdr, and migrate to the anterior side of the embryo. In the CC, Foxa2-vCP progeny contribute to both the FHF and SHF, and are located primarily at the apex of the crescent. Finally, in the differentiated four-chambered heart, Foxa2-vCP progeny give rise to cardiomyocytes, endothelial cells and conduction system cells of the left and right ventricular chambers, and to epicardial cells of the entire heart.

Intriguingly, the newly identified progenitor population expresses *Foxa2*, a gene primarily associated with endoderm and ectoderm development^22^,^23^,^24^. During gastrulation, Foxa2 is expressed in the posterior epiblast underlying the anterior PS^27^. Our data support a model for cardiac development in which Foxa2-vCPs are the first cardiogenic cells that migrate to the anterior side of the embryo during gastrulation, when they comprise approximately half of the CM population. On the anterior side, these progenitors contribute to large parts of the CC structure in a highly defined pattern that overlaps with both FHF and SHF domains. Finally, Foxa2-vCP-derived cells form the majority of cells of both the left and right ventricular chambers, and the epicardium of the entire heart (schematic illustration; Fig. 9).

Our model is in line with recent clonal analysis of *Mesp1*-expressing cardiac progenitor cells, which contribute to the heart in a highly regulated spatiotemporal manner^20^,^57^. One important focus of our study was to investigate the Foxa2-vCP development in the context of the well-characterized FHF and SHF progenitor populations and to determine their developmental and spatial relationship. Our analysis at the CC stage, when FHF and SHF populations occur, illustrates that Foxa2-vCPs contribute to both of these progenitor populations in a defined manner. Specifically, Foxa2-vCPs give rise to the majority of the FHF and to half of the SHF. This correlates with the subsequent contribution of Foxa2-vCPs to primarily the ventricular chambers, as the FHF gives rise primarily to the LV, whereas the SHF gives rise to the RV in addition to other regions of the heart^2^,^10^. As such, lineage tracing of Foxa2-vCPs allows for a new spatial segregation of the CC, which predicts the atrial–ventricular differentiation potential of the cells at this stage during heart development.

The timing of atrial and ventricular specification, as well as the mechanisms by which the two lineages are determined, have been long-standing important questions in the field, as they are crucial for our understanding of the pathogenesis of congenital heart defects and for developing improved strategies for regenerative therapies. Our collective functional analyses have shown that ventricular development is highly sensitive and relies on proper execution of the specification and differentiation programmes. Namely, in the absence of Foxa2, ventricular progenitor specification is compromised and perturbation of these progenitors through either loss of a key member of the cardiac gene regulatory network (Islet1), or modulation of the signalling environment (RA) leads to morphogenetic errors during early heart development. Although functional assays are challenging at these early stages due to possible confounding effects of *Islet1* loss-of-function in endoderm cells, or due to general patterning defects in RA-treated embryos, they nevertheless reveal the investigative power of this new model system and progenitor population. Although ablation of Foxa2 specifically in Foxa2-vCPs would be interesting, the tools to do so are missing. Loss of Foxa2 in the entire embryo has profound effects and renders cardiac-specific studies challenging both due to early lethality of the embryos, as well as to potential confounding effects of other lineages such as endodermal tissues^22^. Foxa2 has further been shown to be expressed in mesendoderm, a population that exists before mesoderm and endoderm divergence^58^. Determining in detail the precise time at which Foxa2 labels Foxa2-vCPs will be pivotal to future functional studies, but remains to be elucidated.

The contribution of Foxa2-vCPs to multiple cell types of both ventricular chambers underscores the potency of this promising new progenitor population. Assessing whether the Foxa2-vCP population segregates into multiple subpopulations determined by distinct spatiotemporal regulatory mechanisms and whether individual cells of the Foxa2-vCP population harbour multi-lineage potential will further contribute to our understanding of the earliest specification events during cardiac development.

Although Foxa2 is no longer present after gastrulation, the lineage tracing of Foxa2-vCPs established here allows for the visualization of the prospective ventricular cells at any stage of development. This represents a unique opportunity to further study atrial–ventricular specification and differentiation by in-depth characterization of this population at key stages during cardiac development and differentiation (CM, CC and early heart tube). Furthermore, gene ablation strategies specifically in the developing and differentiated ventricular lineage are now possible using the *Foxa2Cre* model system, particularly for candidates not involved in endoderm or ectoderm development.

As such, exploring the downstream regulatory mechanisms in the Foxa2-derived ventricular lineage, determined by the expression profiling of mESC-derived Foxa2-expressing CM illustrated here, and by future studies from *in vivo* populations, harbours the tremendous potential to uncover novel regulators of ventricular specification. These mechanisms promise to increase our understanding of congenital heart defects, as well as to enable the establishment of strategies for generating defined cell types from PSCs for the purpose of regenerative medicine and disease modelling.

## Methods

### Mice

The *Foxa2Cre* mouse line (C57BL/6J) was generated and shared with us by Dr Heiko Lickert^28^ and *Islet1*^*floxed*^ (Jax stock number 028501; (129X1/SvJ x 129S1/Sv)F1-Kitl<+>) mice were kindly provided by Dr Sylvia Evans^50^. *Foxa2CreER* (008464), *Rosa-YFP* (006148; C57BL/6J) and *Rosa-tDT* (007914; C57BL/6J) mice were all obtained from The Jackson Laboratory. The Pdgfra-H2B-GFP (007669; 129S4/SvJaeSor) line was a kind gift of Dr Philippe Soriano. For time-course experiments, the day of plug identification corresponds to embryonic day 0.5 (E0.5). For the *Foxa2*-inducible lineage tracing, Tam (Sigma T5648; 50 μg g^−1^) was administered via intraperitoneal injection at days E6.5, E7.5 or 8.5. All-*trans* RA (Sigma R2625) was reconstituted in dimethylsulfoxide (20 mg ml^−1^) and injected at 65 mg kg^−1^ on day E7.5. All animals were housed in facilities operated by the Center for Comparative Medicine and Surgery at Icahn School of Medicine. All animal experiments were conducted in accordance with the guidelines and approval of the Institutional Animal Care and Use Committee at Icahn School of Medicine at Mount Sinai.

### Histology and IF analysis

Embryos were collected from timed pregnancies and either fixed in 4% paraformaldehyde (PFA), washed with PBS and equilibrated in 30% sucrose (Sigma-Aldrich) before embedding or embedded fresh into OCT (Electron Microscopy Sciences). Tissues were subsequently cut into 10 μm sections using a Leica Cryostat. Slides were fixed for 10 min in 4% PFA and incubated for 1 h in blocking solution (PBS with 0.1%Triton and 1% BSA). Primary antibodies were diluted in blocking solution and incubations were carried out for 1 h at room temperature or overnight at 4 °C, followed by incubation in secondary antibody for 1 h at room temperature. Slides were then counterstained with 4,6-diamidino-2-phenylindole and mounted using nPG antifade mounting media.

For WMIF, embryos were collected, immersion-fixed in 4% PFA overnight and washed with PBS. Embryos were blocked for >4 h in blocking solution (PBS with 0.1%Triton and 1% BSA). Primary antibodies were diluted in blocking solution and incubations were carried out overnight at 4 °C. Embryos were washed three times in PBS-T (PBS with 0.1% Triton) for 2 h each, followed by incubation in secondary antibody overnight at 4 °C. Embryos were then counterstained with 4,6-diamidino-2-phenylindole and allowed to equilibrate in nPG antifade mounting media before mounting. Embryos were mounted on uncharged slides using double-stick tape and coverslips. The following primary antibodies and dilutions were used: anti-GFP (Abcam, 1:500), anti-RFP (Rockland, 1:500), anti-cTnT (ThermoSci, 1:300), anti-Endoglin (R&D, 1:100), anti-Wt1 (abcam, 1:100); anti-Mlc2v (Proteintech, 1:100); anti-Foxa2 (Novus, 1:300); anti-Nkx2–5 (Abcam, 1:200, or Santa Cruz, 1:100), anti-Hcn4 (Millipore, 1:100) and anti-Isl1 (Abcam, 1:100). Secondary antibodies conjugated with Alexa dyes were obtained from Jackson Immunoresearch and used at 1:500.

Fluorescence images were obtained using either Leica DM6000 or EVOS slide microscopes. Confocal microscopy was performed using either Leica SP5 DM or Zeiss 780 microscopes. Images were processed using Imaris 8, FIJI ImageJ or Adobe Photoshop software. Surface rendering was performed in Imaris using the Surfaces tool. Specifically, surfaces were generated for reference stain (that is, Nkx2–5) channels. A duplicate YFP channel was then created using the Nkx2–5 surface as a mask and a surface was generated for YFP based on that region. Surface volumes were determined from the statistics panel within Imaris.

### mESC differentiation

mESCs were maintained on Matrigel-coated plates in 2i media^36^. mESCs were differentiated to the cardiomyocyte lineage using an EB differentiation protocol^36^. EBs were generated at day 0 of differentiation in SF-D media (DMEM-F12 75%, Ham's F12 25%, N2 supplement 0.5 × , B27 supplement 0.5 × , BSA 0.05%, L-glutamine 1 × and β-mercaptoethanol 150 μM) with ascorbic acid (0.5 mM) and monothioglycerol (0.4 mM). On day 2, EBs were collected, dissociated and reaggregated in SF-D media with vascular endothelial growth factor (5 ng ml^−1^), Activin A (8 ng ml^−1^) and BMP4 (1 ng ml^−1^) to induce CM formation. At day 5 EBs were collected, washed and cultured in StemPro-34 (Gibco) with vascular endothelial growth factor (5 ng ml^−1^), basic fibroblast growth factor (10 ng ml^−1^) and XAV (10 μM) to induce cardiomyocyte differentiation. Subsequent media changes were done in the same media without XAV.

For the generation of endoderm cells from mESCs, EBs were generated at day 0 of differentiation in SF-D media. On day 2, EBs were collected, dissociated and reaggregated in SF-D media with Activin A (75 ng ml^−1^) to induce endoderm formation by day 5 (ref. 42).

### Mixed chimera competition assay

*WT* tdT and *Foxa2−/−* LynTomato mESCs were dissociated to single cells with TrypLE. Cells were washed with PBS and stored in media at 37 °C until injection into hosts. WT mouse blastocysts were collected from pregnant females at day E3.5 just before injection. 10–15 mESCs were injected into the each blastocyst cavity near the inner cell mass of the embryo. After injection, 12 blastocysts were implanted into each female and allowed to develop until day E13.5, when the embryos were harvested for analysis.

### Flow cytometry analysis and cell sorting

*Cells*. EBs generated from mESC differentiation cultures or E7.25–8.25 embryos were dissociated with TrypLE or 0.25% Trypsin/EDTA, respectively. Cells were washed in staining solution (DMEM with 0.1% BSA), pelleted and resuspended in staining solution. Antibodies were diluted in staining solution and cells were incubated on ice for 30 min. Cells were then washed, filtered and resuspended in staining solution for analysis or cell sorting.

*Tissues*. For flow cytometry analysis of atrial and ventricular cells, dissected hearts were dissociated and dissected into atrial and ventricular portions. Hearts were minced using a scalpel and incubated for 1 h in collagenase II (Worthington), washed with PBS, incubated with TrypLE for 30 min, washed with PBS and stained with appropriate antibodies in staining solution.

Cell counts were collected using a LSRII (BD Biosciences) and data were analysed using the FlowJo software. The following antibodies and dilutions were used: anti-Pdgfra-BV421 (BD, 1:100); anti-Kdr-PE-Cy7 (BD, 1:100); anti-hCD4-PE (Life Technologies, 1:100); anti-Epcam (eBioscience, 1:100); anti-cTnT (ThermoSci, 1:100); CD90 (Biolegend, 1:100); CD45 (BD, 1:100); CD31 (BD, 1:100); Ter119 (BD, 1:100).

### Real-time quantitative PCR

Total RNA was prepared with the RNAqueous-Micro Kit (Ambion) and treated with RNase-free DNase (Ambion). Reverse transcription was performed from 100 to 500 ng of RNA using the Quanta qScript kit. Quantitative PCR was carried out on an Applied Biosystems Step One Plus using ABI SYBR Green reagents. Expression levels were normalized to β-actin. In addition, for normalization across samples genomic DNA was used to generate a standard curve^59^. The *y* axis of reverse transcriptase–quantitative PCR graphs represents copy numbers of the gene of interest divided by copy numbers of β-actin and therefore is an arbitrary but absolute unit that can be compared between experiments.

### Complementary DNA library preparation and RNAseq

Total RNA obtained from fluorescent activated cell-sorted cells was purified with the RNAqueous Micro kit (Life Technologies) and quantified by NanoDrop spectrophotometer (Thermo Scientific) or Qubit fluorimeter (Life Technologies). RNA was assessed for quality using the Agilent Bioanalyzer. Samples with RNA integrity number scores ≥9 were further processed. Sample cDNA libraries were prepared using the Illumina TruSeq RNA v2 kit and 500 ng of input. Library concentration and quality was quantified by Qubit and Bioanalyzer and subsequently sequenced on the Illumina HiSeq 2500 platform using a 50 nt single-end read setting.

### RNAseq data processing

Quality analysis of raw sequencing reads was performed using FastQC ( http://www.bioinformatics.bbsrc.ac.uk/projects/fastqc/). Reads were then aligned to the mouse reference genome (mm10) using STAR with the two-pass setting^60^ and a database of known splice junctions from the ENCODE mm10/GRCm38 annotation for the initial alignment ( http://www.gencodegenes.org/mouse_releases/2.html). Picard tools ( http://broadinstitute.github.io/picard/) was used to index and remove duplicate reads from the resulting SAM files, generating 16–25 million unique aligned reads per sample. From the resulting alignments, we used HTSeq^61^ to generate read counts per transcript, and normalized these and performed differential expression analysis using DESeq2 (ref. 62). Gene Ontology analysis was performed using Panther GO ( http://geneontology.org/page/go-enrichment-analysis). Chord plot was generated using the R package GOplot (v 1.0.1). All heatmaps were generated using the R package pheatmap (v 1.0.8). Principle component analysis and mean average (PCA and MA) plots were generated using the R packages ggplot2 (v 2.0.0) and DESeq2 (v 1.10.1). The RNAseq data have been deposited in the NCBI database (see details below).

### Data availability

The authors declare that all data supporting the findings of this study are available within the article and its Supplementary Information files, or from the corresponding author upon reasonable request. The RNAseq data have been deposited in the NCBI GEO database under accession code GSE78964.

## Additional information

**How to cite this article:** Bardot, E. *et al*. Foxa2 identifies a cardiac progenitor population with ventricular differentiation potential. *Nat. Commun.* **8,** 14428 doi: 10.1038/ncomms14428 (2017).

**Publisher's note:** Springer Nature remains neutral with regard to jurisdictional claims in published maps and institutional affiliations.

## Supplementary Material

Supplementary Information Supplementary Figures.

Supplementary Movie 1 3D volume of confocal z-projection of E8.25 Foxa2Cre:YFP embryo analysed by whole mount immunofluorescence (WMIF) using antibodies against YFP (green), Nkx2-5 (red), and Hcn4 (blue). Data reflects z-projections shown in Figure 4b.

Supplementary Movie 2 Confocal z-stack animation of whole mount immunofluorescence of E8.25 Foxa2Cre:YFP embryo using antibodies against YFP (green), Nkx2-5 (red), and Hcn4 (blue). Slices are shown in a posterior to anterior direction. Data reflects z-projections shown in Figure 4b.

Supplementary Movie 3 3D volume of confocal z-projection of E8.25 Foxa2Cre:YFP embryo analysed by whole mount immunofluorescence (WMIF) using antibodies against YFP (green), Nkx2-5 (red), and Isl1 (blue). Data reflects z-projections shown in Figure 4d.

Supplementary Movie 4 Confocal z-stack animation of whole mount immunofluorescence of E8.25 Foxa2Cre:YFP embryo using antibodies against YFP (green), Nkx2-5 (red), and Isl1 (blue). Slices are shown in a posterior to anterior direction. Data reflects z-projections shown in Figure 4d and Supplementary Figure 9c.

Supplementary Movie 5 High magnification confocal z-stack animation of whole mount immunofluorescence of E8.25 Foxa2Cre:YFP embryo using antibodies against YFP (green), Nkx2-5 (red), and Isl1 (blue). Slices are shown in a posterior to anterior direction. Data reflects z-projections shown in Supplementary Figure 9a, b.

Supplementary Movie 6 3D surface rendering generated from confocal z-projeciton of whole mount immunofluorescence of E8.25 Foxa2Cre:YFP embryo using antibodies against YFP (green), Nkx2-5 (red), and Hcn4 (blue). 3D surfaces were generated for Nkx2-5 and Hcn4 and the Hcn4 surface was then used to mask the YFP signal before generating the YFP surface. The YFP surface thus reflects only YFP signal within the Hcn4+ region. Note that presented colors do not indicate channel merges. Data reflects z-projections shown in Figure 4c.

Supplementary Movie 7 3D surface rendering generated from confocal z-projeciton of whole mount immunofluorescence of E8.25 Foxa2Cre:YFP embryo using antibodies against YFP (green), Nkx2-5 (red), and Isl1 (blue). 3D surfaces were generated for Nkx2-5 and Isl1 and the Isl1 surface was then used to mask the YFP signal before generating the YFP surface. The YFP surface thus reflects only YFP signal within the Isl1+ region. Note that presented colors do not indicate channel merges. Data reflects z-projections shown in Figure 4e.

Supplementary Movie 8 3D volume of confocal z-projection of E8.5 Foxa2Cre:YFP embryo analysed by whole mount immunofluorescence (WMIF) using antibodies against YFP (green), cTnT (red), and Isl1 (blue). Data reflects z-projections shown in Figure 4i.

Supplementary Movie 9 3D surface rendering generated from confocal z-projeciton of whole mount immunofluorescence of E8.5 Foxa2Cre:YFP embryo using antibodies against YFP (green), cTnT (red), and Isl1 (blue). 3D surfaces were generated for cTnT and Isl1. Two YFP surfaces were generated: one from the total signal, and a second using the cTnT surface to mask the YFP signal before generating the YFP surface. The YFP surface shown starting at 0:07 thus reflects only YFP signal within the heart tube region. Note that presented colors do not indicate channel merges. Data reflects z-projections shown in Figure 4i.

Supplementary Movie 10 3D volume of confocal z-projection of E9.5 WT embryo analysed by whole mount immunofluorescence (WMIF) using antibodies against cTnT (green), and Nkx2-5 (red). Data reflects z-projections shown in Figure 8d.

Supplementary Movie 11 3D surface rendering generated from confocal z-projection of E9.5 WT embryo analysed by whole mount immunofluorescence (WMIF) using antibodies against cTnT (green). Data reflects z-projections shown in Figure 8d.

Supplementary Movie 12 3D volume of confocal z-projection of E9.5 Foxa2Cre:Isl1lox/lox embryo analysed by whole mount immunofluorescence (WMIF) using antibodies against cTnT (green), and Nkx2-5 (red). Data reflects z-projections shown in Figure 8d.

Supplementary Movie 13 3D surface rendering generated from confocal z-projection of E9.5 Foxa2Cre:Isl1lox/lox embryo analysed by whole mount immunofluorescence (WMIF) using antibodies against cTnT (green). Data reflects z-projections shown in Figure 8d.
