# Supplementary material for: Foxa2 identifies a cardiac progenitor population with ventricular differentiation potential
Source: Nat Commun. 2017 Feb 14;8:14428. doi: 10.1038/ncomms14428 (PMC5316866; doi:10.1038/ncomms14428)
Supplement: Supplementary Information — Supplementary Figures. [file ncomms14428-s1.pdf]

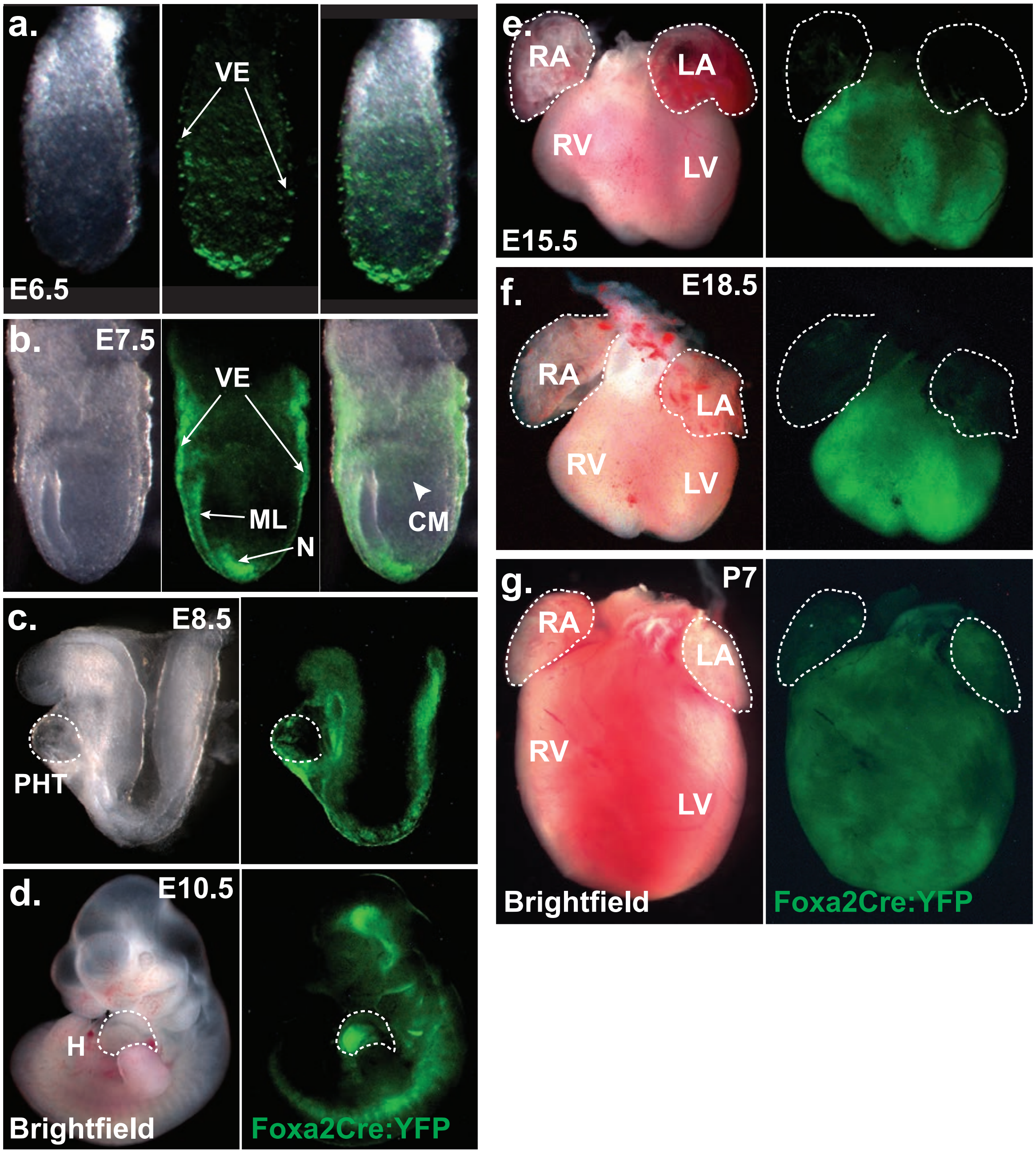
a.
E6.5
VE
b.
E7.5
VE
ML
N
CM
c.
E8.5
PHT
d.
E10.5
H
Brightfield
Foxa2Cre:YFP
e.
RA
LA
RV
LV
E15.5
f.
E18.5
RA
LA
RV
LV
g.
P7
RA
LA
RV
LV
Brightfield
Foxa2Cre:YFP

Supplementary Figure 1: Lineage tracing of Foxa2-expressing cells during mouse development. Whole mount imaging of live *Foxa2Cre* lineage tracing embryos (a-d) or dissected hearts (e-g). *Foxa2Cre* embryos from crosses with *R26-YFP* (YFP, green) are shown in lateral view. Dashed lines outline the heart tube (c-d) or the atrial chambers (e-g). *A, anterior; P, posterior; ML, midline; N, node; CM, cardiac mesoderm; PHT, primitive heart tube; H, heart; RA, right atria; RV, right ventricle; LA, left atria; LV, left ventricle.*

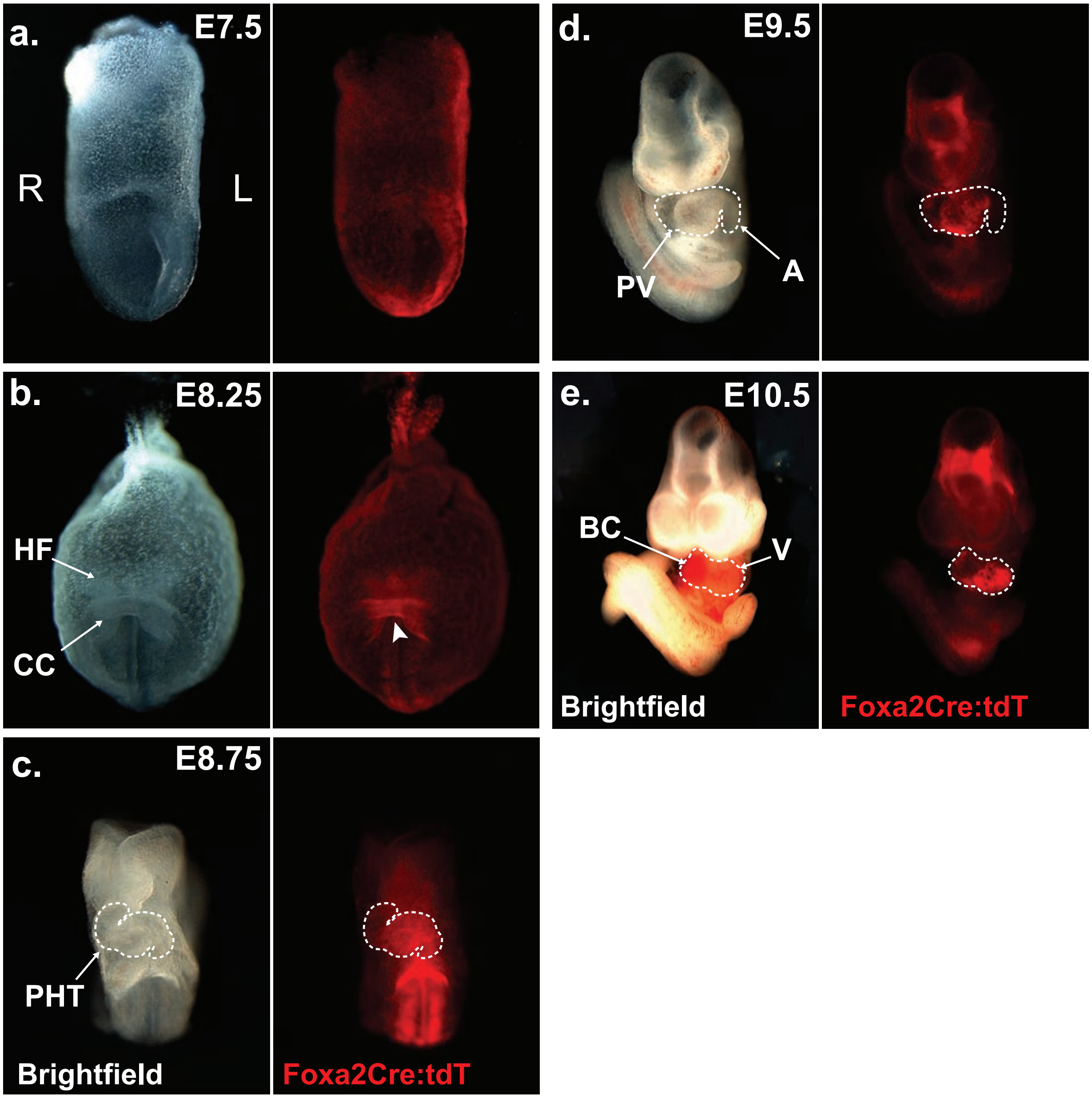
a.
E7.5
R
L
b.
E8.25
HF
CC
c.
E8.75
PHT
Brightfield
Foxa2Cre:tdT
d.
E9.5
PV
A
e.
E10.5
BC
V
Brightfield
Foxa2Cre:tdT

Supplementary Figure 2: Lineage tracing of Foxa2-expressing cells during mouse development. Whole mount imaging of live *Foxa2Cre* lineage tracing embryos (a-d) or dissected hearts (e-g). *Foxa2Cre* embryos from crosses with *R26-tdT* (tdT, red) are shown in en face view. Dashed lines outline the heart tube (c-e). *R, right; L, left; ML, midline; N, node; CM, cardiac mesoderm; HF, head folds; CC, cardiac crescent; PHT, primitive heart tube; PV, primitive ventricle; A, atria; BC, bulbis cordis; V, ventricle.*

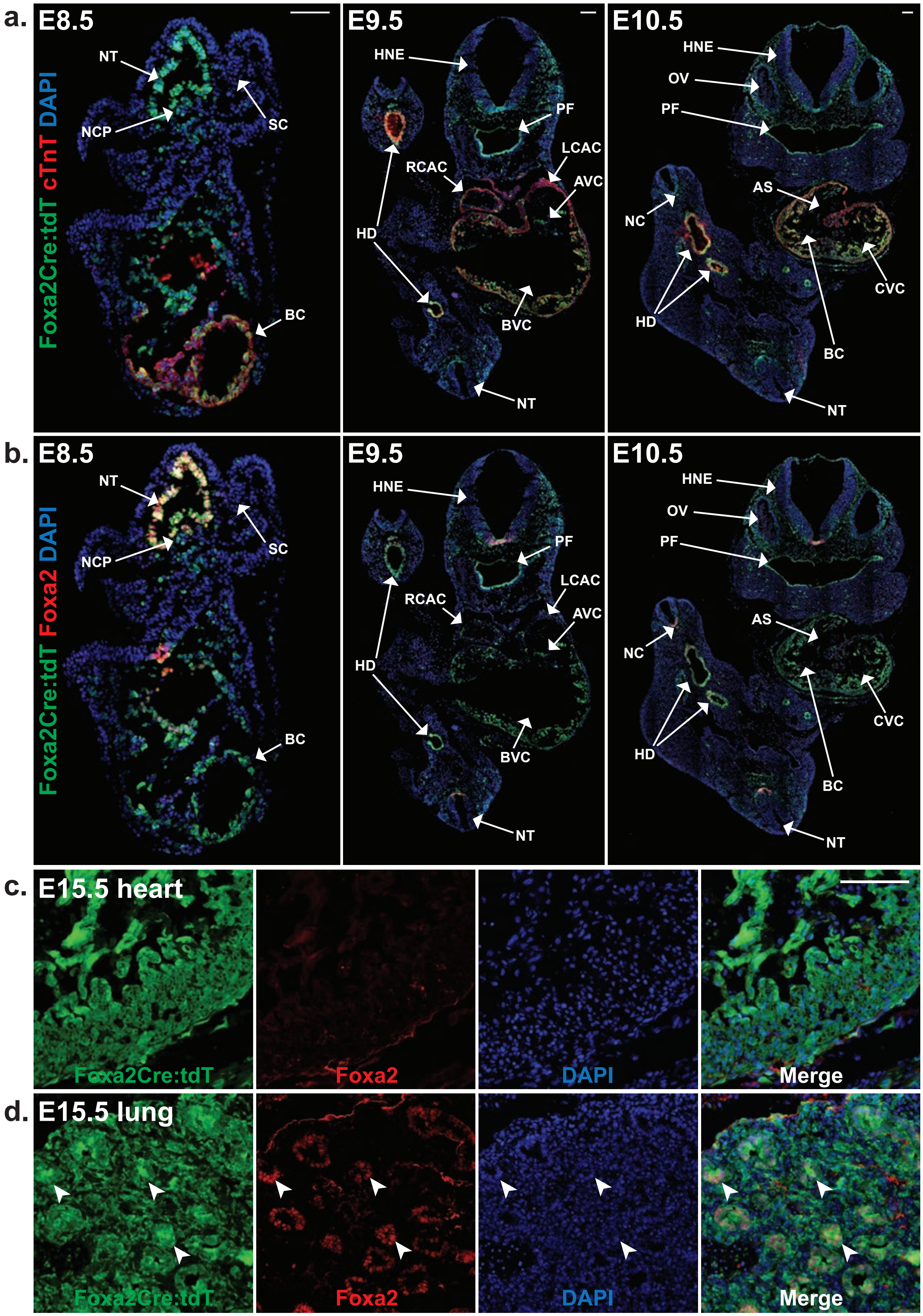
a.
E8.5
Foxa2Cre:tdT cTnT DAPI
NT
NCP
SC
BC
E9.5
HNE
PF
LCAC
RCAC
AVC
HD
BVC
NT
E10.5
HNE
OV
PF
AS
NC
HD
CVC
BC
NT
b.
E8.5
Foxa2Cre:tdT Foxa2 DAPI
NT
NCP
SC
BC
E9.5
HNE
PF
LCAC
RCAC
AVC
HD
BVC
NT
E10.5
HNE
OV
PF
AS
NC
HD
CVC
BC
NT
c.
E15.5 heart
Foxa2Cre:tdT
Foxa2
DAPI
Merge
d.
E15.5 lung
Foxa2Cre:tdT
Foxa2
DAPI
Merge

Supplementary Figure 3: Lineage tracing of Foxa2-expressing cells during mouse development. (a-b) Immunofluorescence analysis of cryosectioned embryos at E8.5 (left), E9.5 (middle), and E10.5 (right) with antibodies against tdT (pseudocolored green) and cTnT (a) or Foxa2 (b). **(c-d)** Immunofluorescence of E15.5 heart (c) or lung (d) sections with antibodies against tdT (pseudocolored green) and Foxa2. *NT, neural tube; NCP, notochordal plate; SC, somatic condensation; BC, bulbis cordis; HNE, hindbrain neuroepithelium; HD, hindgut diverticulum; PF, pharyngeal region of foregut; RCAC, right common atrial chamber; LCAC, left common atrial chamber; AVC, atrioventricular canal; BVC, bulboventricular canal; NT, neural tube; OV, otic vesicle; AS, aortic sac; CVC, common ventricular chamber; NC, notochord.* White arrowheads indicate coexpression of Foxa2 and tdT in the lung epithelium. Scale bars are 100um.

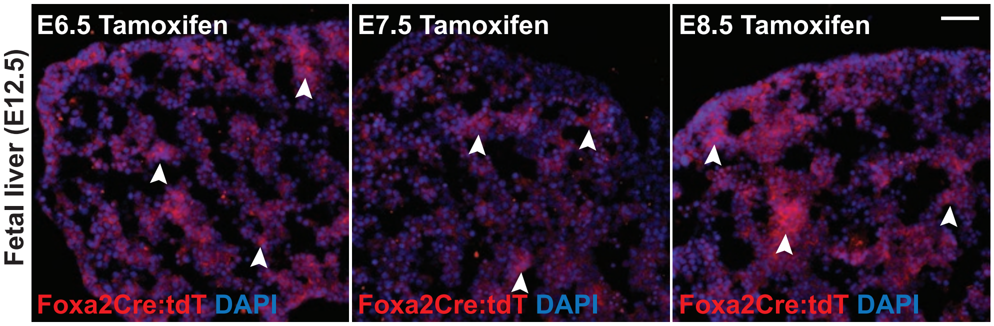
Fetal liver (E12.5)
E6.5 Tamoxifen
E7.5 Tamoxifen
E8.5 Tamoxifen
Foxa2Cre:tdT DAPI
Foxa2Cre:tdT DAPI
Foxa2Cre:tdT DAPI

Supplementary Figure 4: Inducible Foxa2 lineage tracing reveals continuous expression in the fetal liver irrespective of the time of induction. Liver sections from E12.5 Foxa2CreER:tdT embryos injected with tamoxifen at E6.5 (left), E7.5 (middle), or E8.5 (right) were stained with antibodies against tdT. Scale bar is 100um.

Supplementary Figure 5

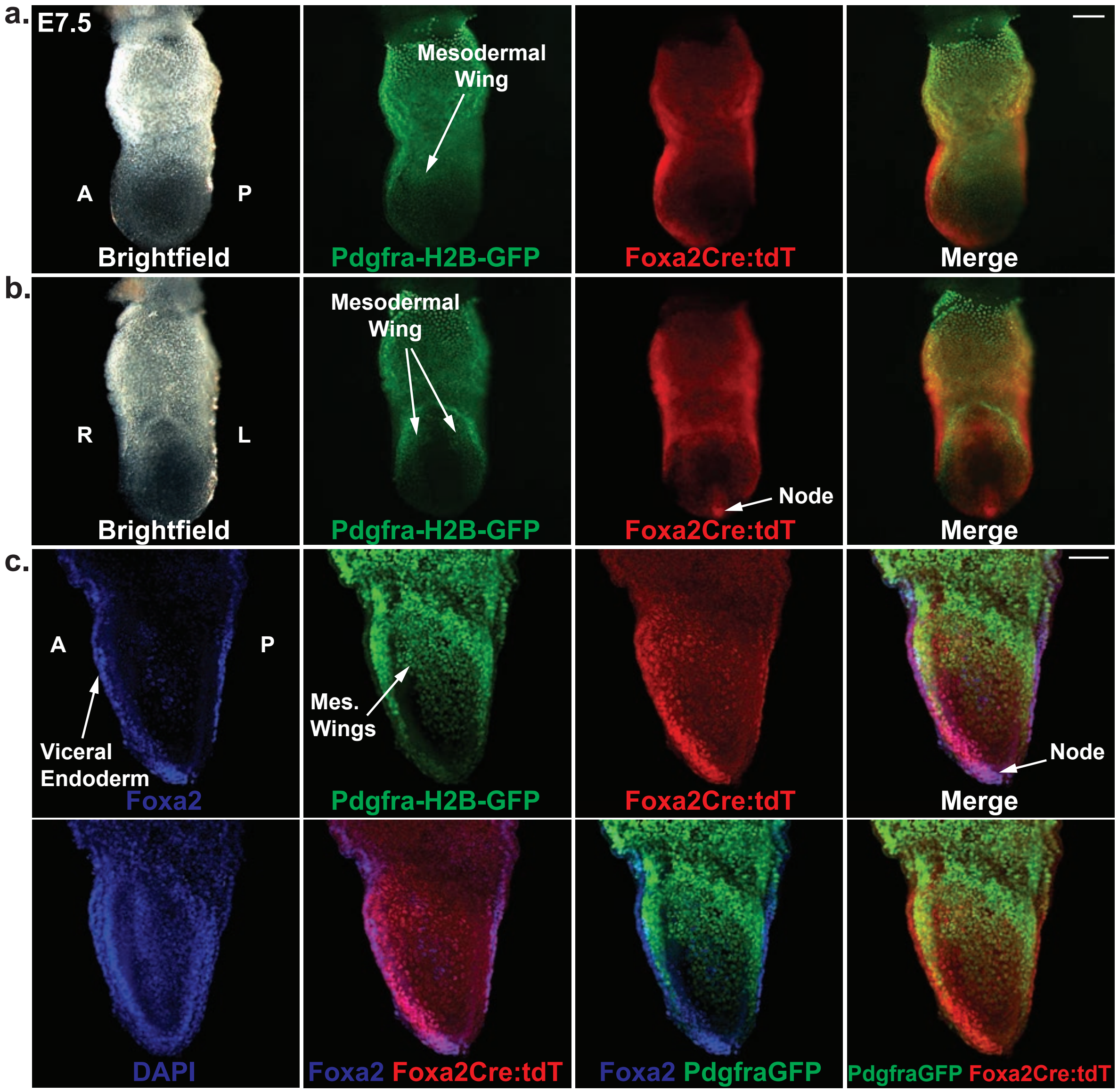

Supplementary Figure 5: *Foxa2* expression marks a transient progenitor population during gastrulation that gives rise to cardiac mesoderm. (a-b) Whole mount live images of E7.5 *Foxa2Cre:tdT Pdgfra-H2B-GFP* embryo in lateral (a) or *en face* (b) views. **(c)** Whole mount immunofluorescence and confocal imaging of E7.5 *Foxa2Cre:tdT Pdgfra-H2B-GFP* embryo stained for GFP, tdT, and Foxa2 protein. *A, anterior; P, posterior; R, right; L, left.* Scale bars are 100um.

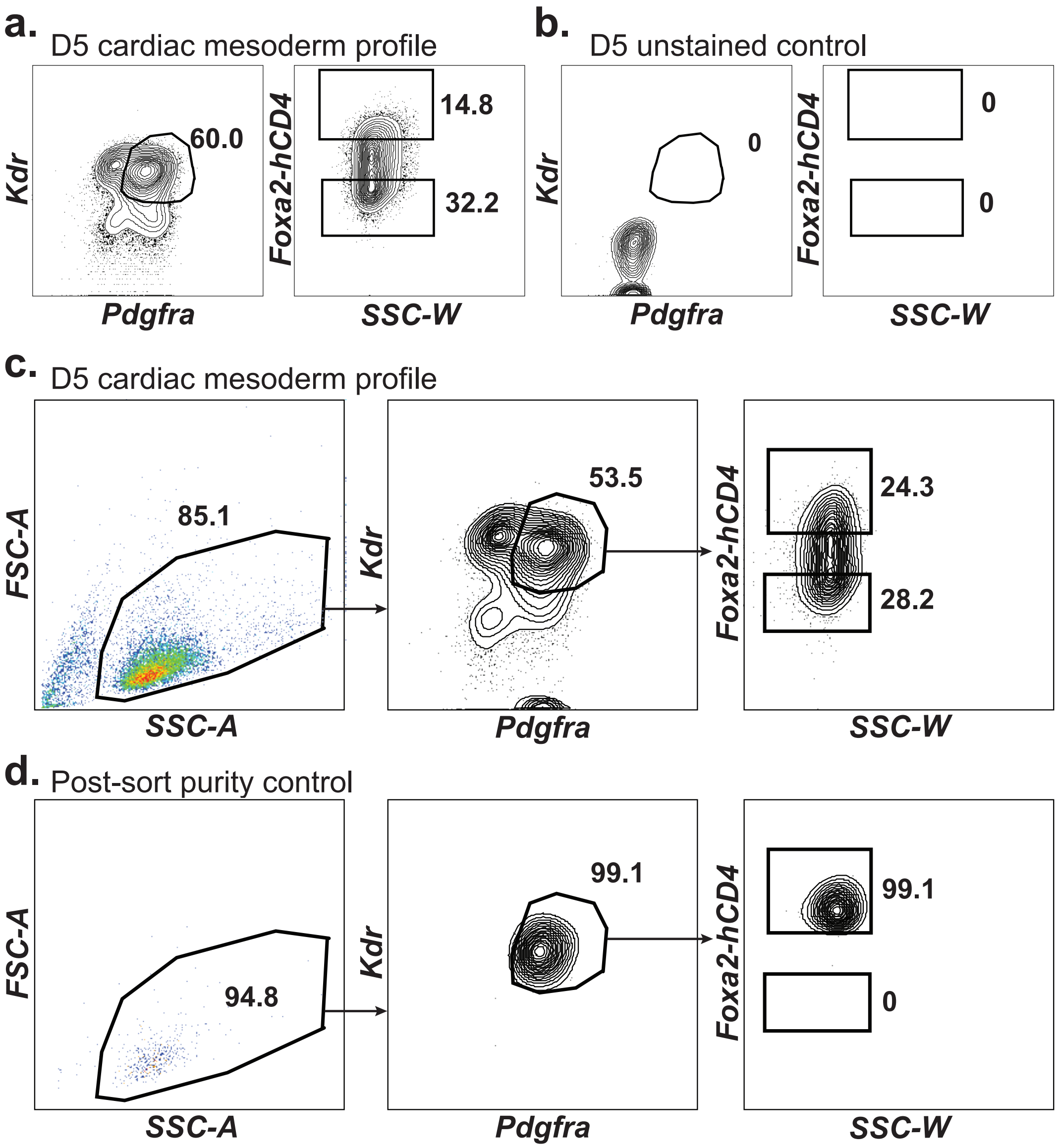
a.
D5 cardiac mesoderm profile
Kdr
Pdgfra
60.0
Foxa2-hCD4
SSC-W
14.8
32.2
b.
D5 unstained control
Kdr
Pdgfra
0
Foxa2-hCD4
SSC-W
0
0
c.
D5 cardiac mesoderm profile
FSC-A
SSC-A
85.1
Kdr
Pdgfra
53.5
Foxa2-hCD4
SSC-W
24.3
28.2
d.
Post-sort purity control
FSC-A
SSC-A
94.8
Kdr
Pdgfra
99.1
Foxa2-hCD4
SSC-W
99.1
0

Supplementary Figure 6: mESC differentiation and isolation of Foxa2+ cardiac mesoderm. (a) Flow cytometric analysis of characteristic cardiac mesoderm profile at day 5 of differentiation. Cardiac mesoderm is labeled by Kdr and Pdgfra, and is further gated based on Foxa2-hCD4 expression. **(b)** Unstained control showing specificity of antibodies used to label cardiac mesoderm populations. **(c)** Fluorescence activated cell sorting (FACS) gating strategy to isolate Foxa2+ and Foxa2- cardiac mesoderm at day 5 of differentiation. **(d)** Foxa2+ cardiac mesoderm was used for post-sort purity analysis via FACS.

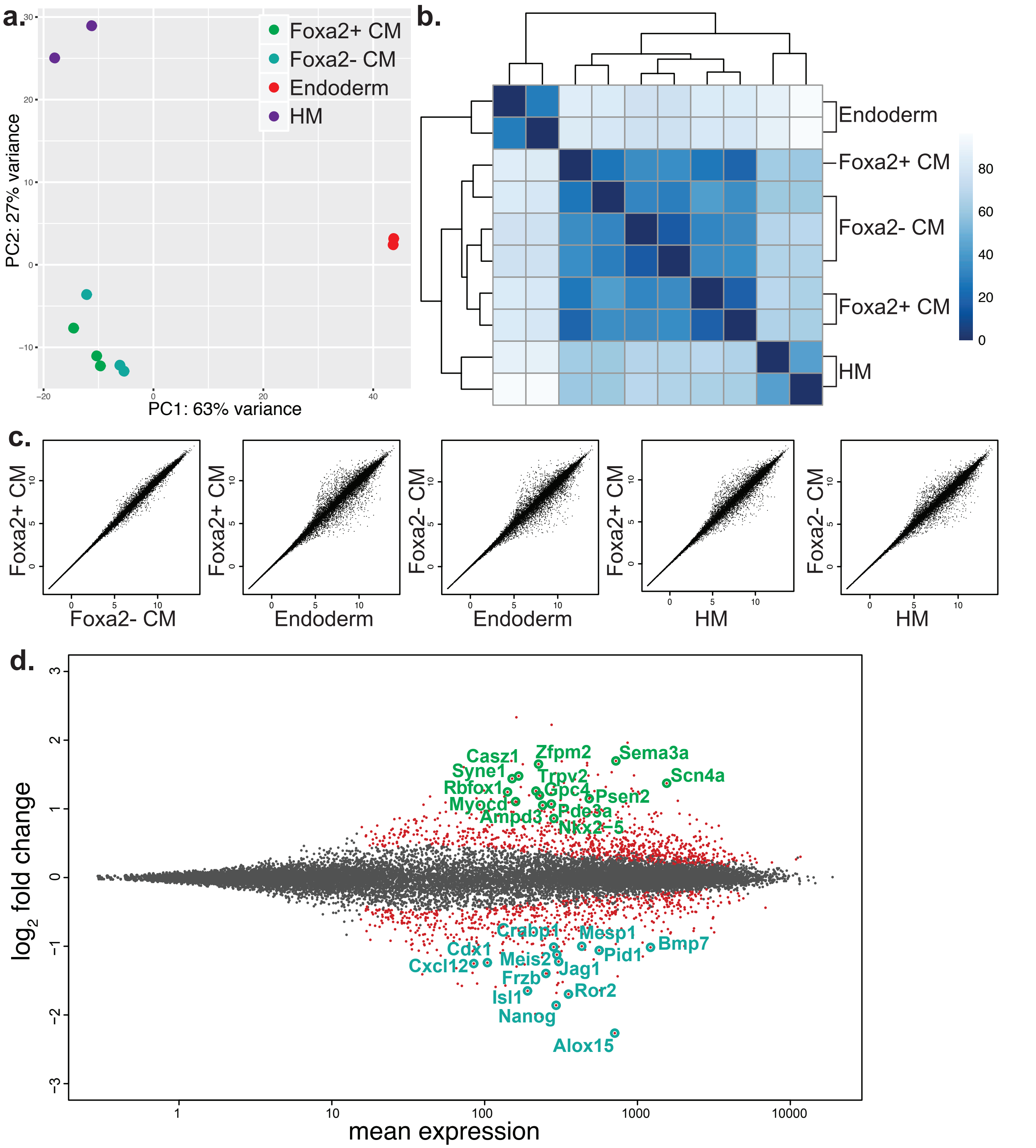
a.
Foxa2+ CM
Foxa2- CM
Endoderm
HM
PC2: 27% variance
PC1: 63% variance
b.
Endoderm
Foxa2+ CM
Foxa2- CM
Foxa2+ CM
HM
c.
Foxa2+ CM
Foxa2- CM
Foxa2+ CM
Endoderm
Foxa2- CM
Endoderm
Foxa2+ CM
HM
Foxa2- CM
HM
d.
log2 fold change
mean expression
Casz1
Zfpm2
Sema3a
Syne1
Trpv2
Scn4a
Rbfox1
Gpc4
Psen2
Myocd
Ampd3
Pde3a
Nkx2-5
Crabp1
Mesp1
Cdx1
Pid1
Bmp7
Cxcl12
Meis2
Jag1
Frzb
Isl1
Ror2
Nanog
Alox15

Supplementary Figure 7: Characterization of a Foxa2+ cardiac mesoderm population during mESC differentiation. (a) Principle Component Analysis of individual RNAseq samples. **(b)** Clustered heatmap of Euclidian distances between each RNAseq sample. **(c)** Scatterplots comparing rlog transformed read counts for one representative replicates from each population. **(d)** MA plot showing log2 fold change of CMF+ over CMF- versus mean expression for three replicates. Significantly expressed genes are shown by red points (Benjamini Hochberg corrected P value <0.05). Some key differentially expressed genes of interest are indicated (upregulated in CMF+, green; downregulated, teal).

Bardot et al., 2016 **Supplementary Figure 8**

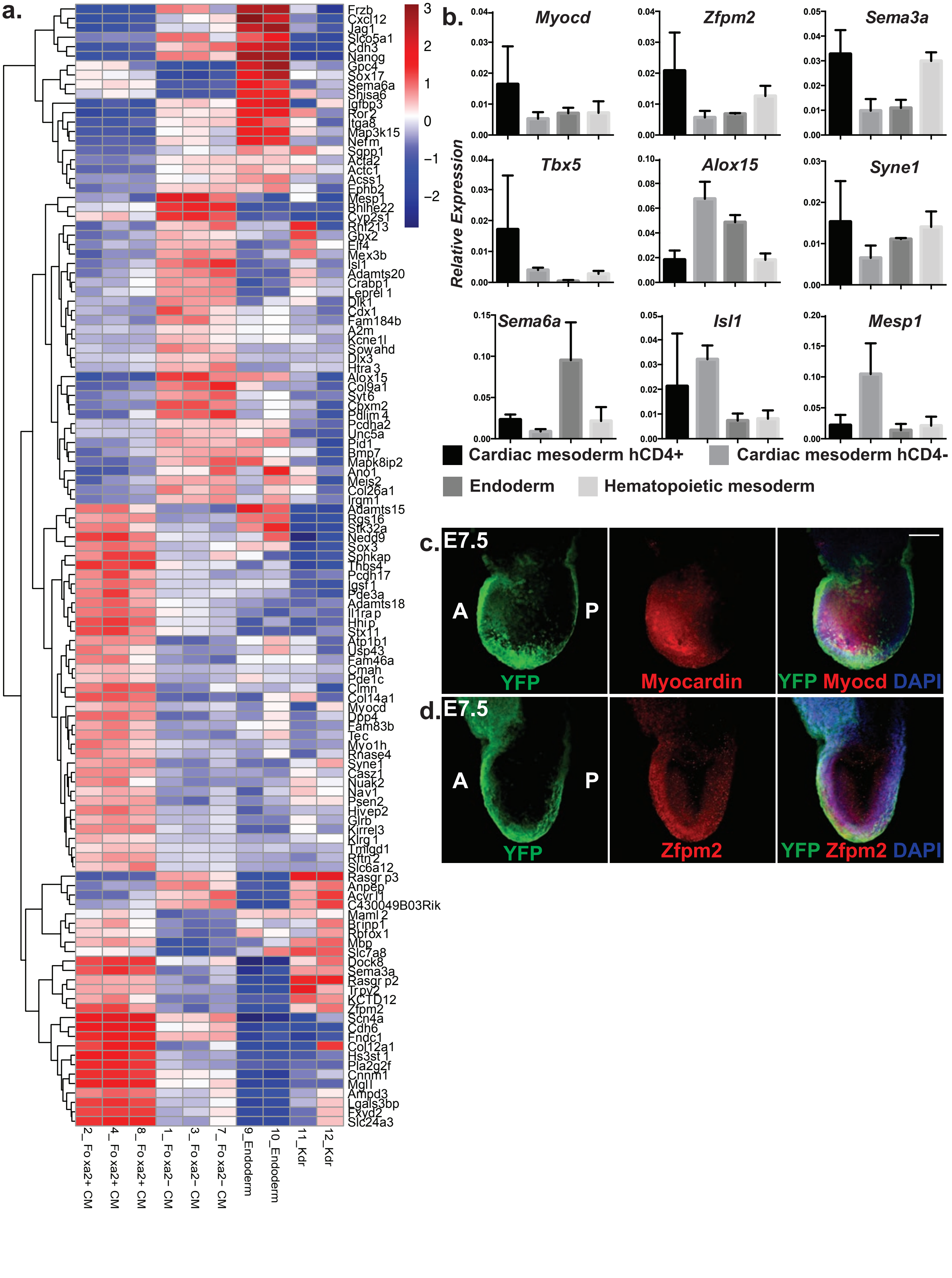

Supplementary Figure 8: Differential gene expression in mESC-derived mesoderm and endoderm populations. (a) Heatmap of log2 fold change in expression relative to the mean for significantly differentially expressed genes across sorted populations (Benjamini Hochberg corrected P value <0.05, log2 fold change >1 or <-1). **(b)** Validation of RNA-seq data by RT-qPCR. Gene expression is shown relative to B-actin in arbitrary units. Data are mean ± SD, n=3 differentiations. **(c-d)** Validation of RNA-seq data by WMIF analysis of E7.5 *Foxa2Cre:YFP* embryos for Myocardin (c) or Zfpm2 (d). Scale bar is 100um.

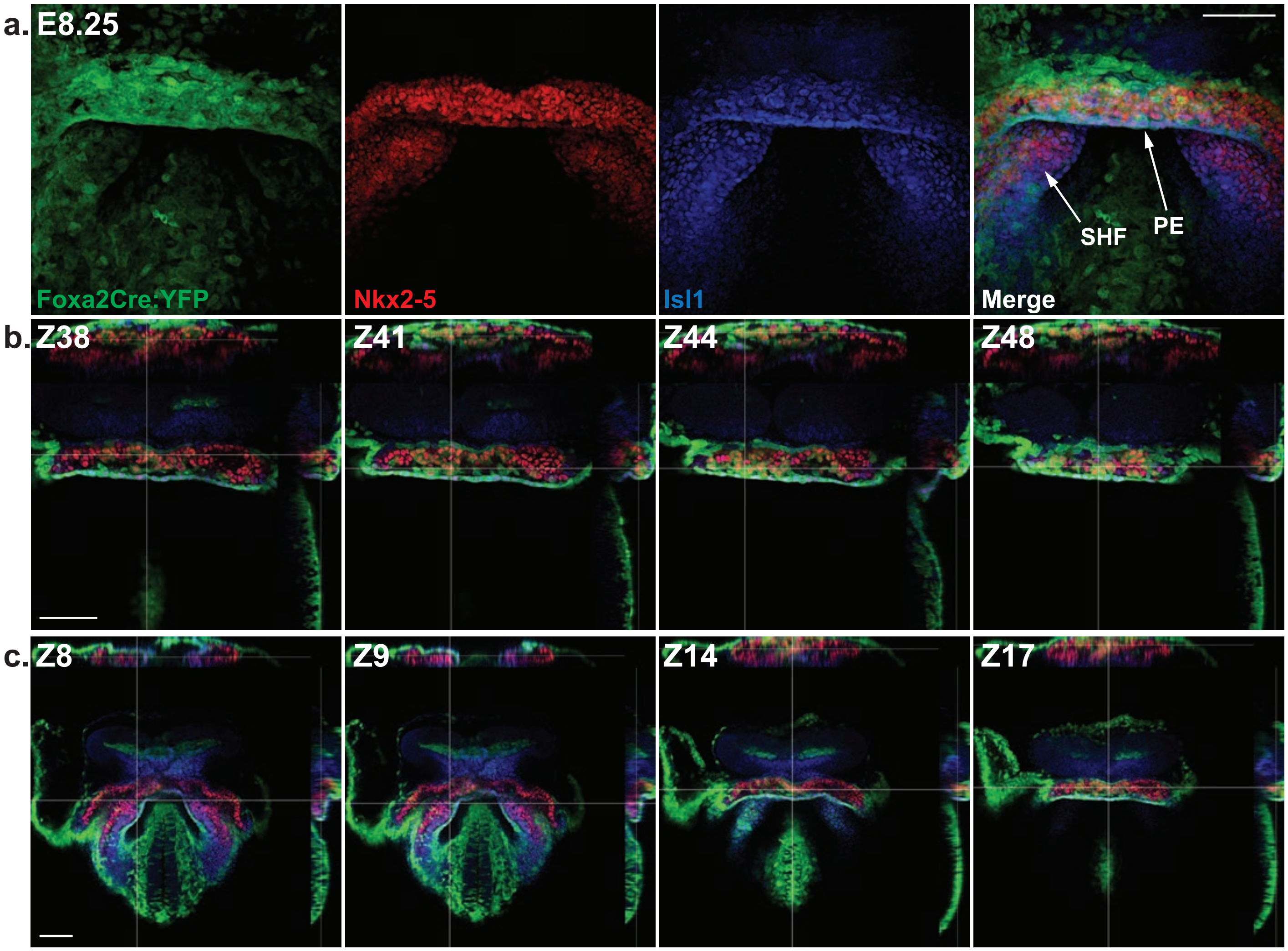
a.
E8.25
Foxa2Cre:YFP
Nkx2-5
Isl1
SHF
PE
Merge
b.
Z38
Z41
Z44
Z48
c.
Z8
Z9
Z14
Z17

Supplementary Figure 9: Foxa2-vCPs contribute to the first and second heart fields during heart morphogenesis. (a) WMIF analysis of E8.25 *Foxa2Cre:YFP* embryo with antibodies against Isl1 and Nkx2-5. High magnification image of data shown in Figure 4c. **(b-c)** Orthogonal views of data in (a) (b) or in Figure 4c (c). Scale bars are 100um.

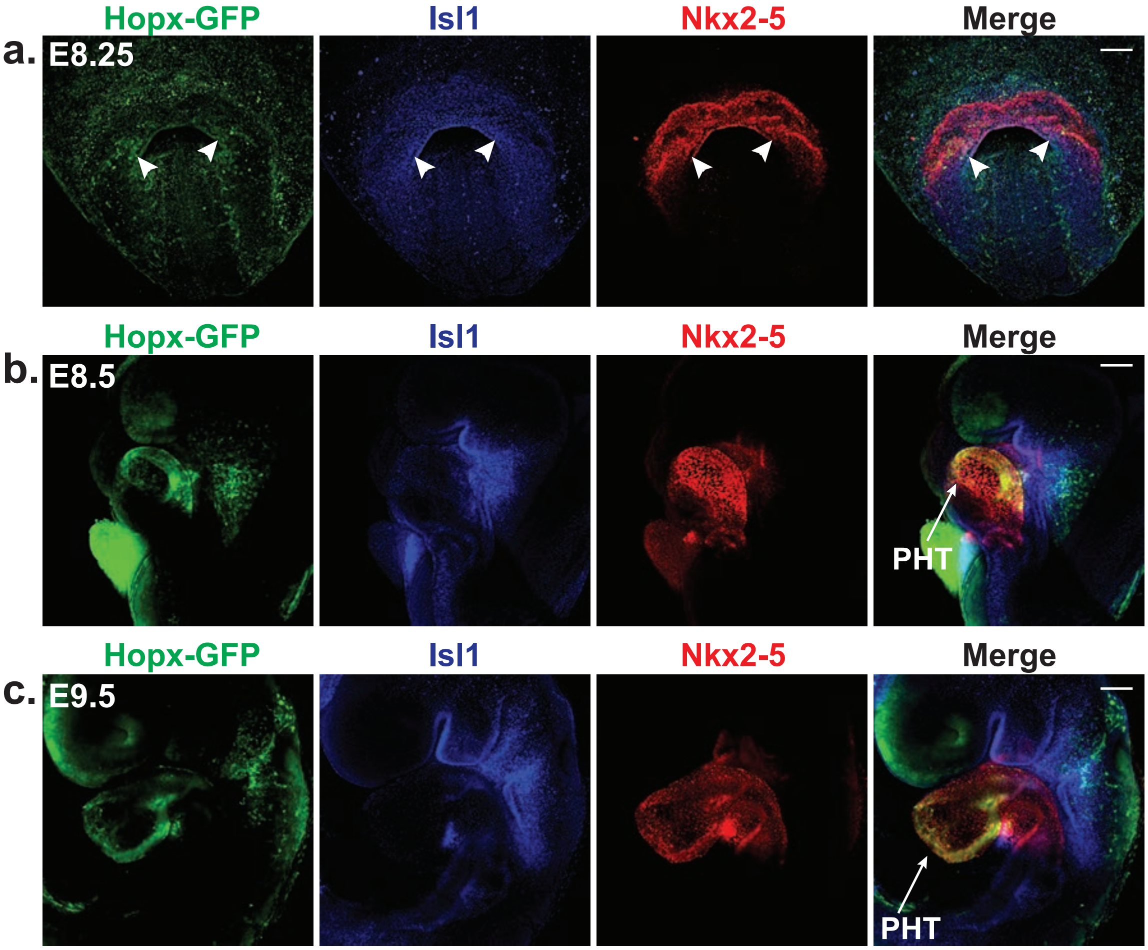
Hopx-GFP
Isl1
Nkx2-5
Merge
a.
E8.25
b.
E8.5
PHT
c.
E9.5
PHT

Supplementary Figure 10: Foxa2-vCPs migration to the first and second heart fields during heart morphogenesis precedes Hopx upregulation. (a-c) WMIF analysis of *Hopx-GFP* embryos at E8.25 (a), E8.5 (b), or E9.5 (c) with antibodies against GFP, Isl1, and Nkx2-5. Arrowheads indicate the presence of few Hopx-GFP+ cells at E8.25. *PHT, primitive heart tube.* Scale bars are 100um.

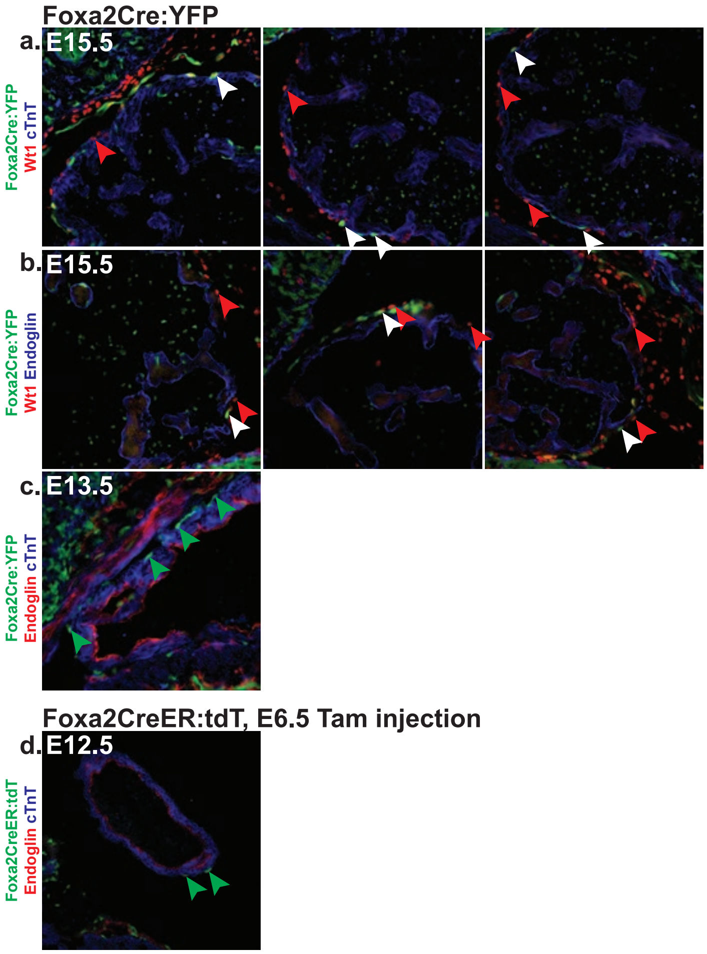
Foxa2Cre:YFP
a.
E15.5
Foxa2Cre:YFP Wt1 cTnT
b.
E15.5
Foxa2Cre:YFP Wt1 Endoglin
c.
E13.5
Foxa2Cre:YFP Endoglin cTnT
Foxa2CreER:tdT, E6.5 Tam injection
d.
E12.5
Foxa2CreER:tdT Endoglin cTnT

Supplementary Figure 11: Contribution of Foxa2-vCPs to the differentiated lineages in the heart. (a) Immunofluorescence analysis of E15.5 Foxa2Cre:YFP embryos using antibodies against YFP, Wt1, and cTnT. (b) Immunofluorescence analysis of E15.5 Foxa2Cre:YFP embryos using antibodies against YFP, Wt1, and Endoglin. (c) Immunofluorescence analysis of E13.5 Foxa2Cre:YFP embryos using antibodies against YFP, Endoglin, and cTnT. (d) Immunofluorescence analysis of E12.5 Foxa2CreER:YFP embryos following tamoxifen induction at E6.5, using antibodies against YFP, Endoglin, and cTnT. Arrowheads indicate cells that express the relevant lineage markers alone (red), YFP alone (green), or that co-express YFP and the red lineage marker (white).

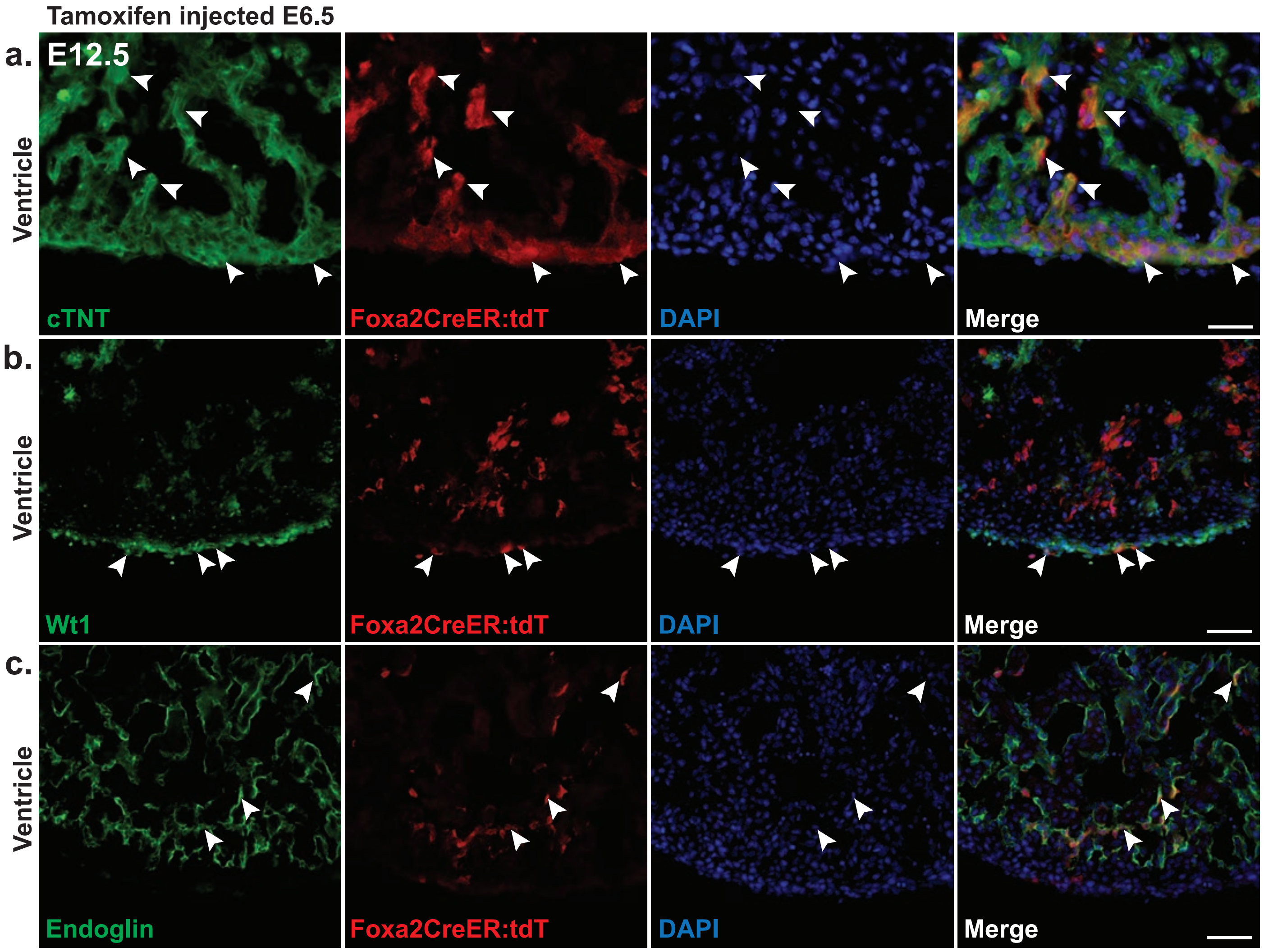
Tamoxifen injected E6.5
a.
E12.5
Ventricle
cTNT
Foxa2CreER:tdT
DAPI
Merge
b.
Ventricle
Wt1
Foxa2CreER:tdT
DAPI
Merge
c.
Ventricle
Endoglin
Foxa2CreER:tdT
DAPI
Merge

Supplementary Figure 12: Inducible *Foxa2* lineage tracing reveals the emergence of a Foxa2+ progenitor population during gastrulation. (a-c) Heart sections from E12.5 *Foxa2CreER:tdT* embryos injected with tamoxifen at E6.5 were stained with antibodies against tdT and either cTnT (a), Wt1 (b), or Endoglin (c). Arrowheads indicate cells that co-express YFP and the relevant markers (white). Scale bars are 50um.

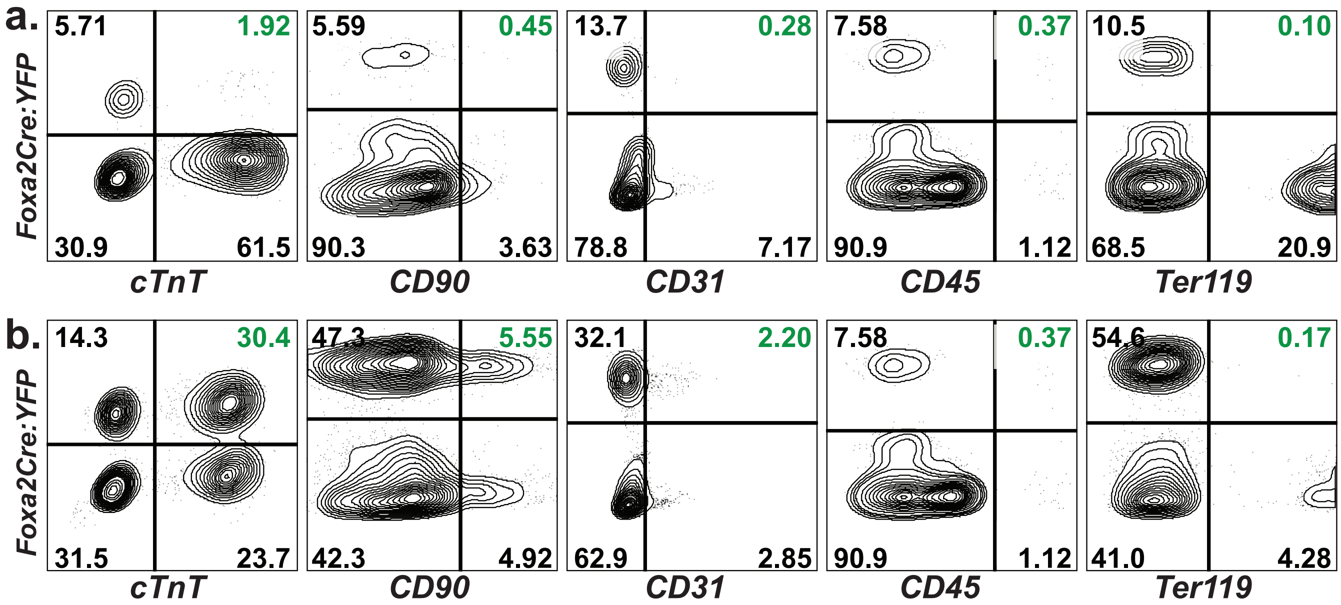
a.
5.71
1.92
30.9
61.5
cTnT
5.59
0.45
90.3
3.63
CD90
13.7
0.28
78.8
7.17
CD31
7.58
0.37
90.9
1.12
CD45
10.5
0.10
68.5
20.9
Ter119
Foxa2Cre:YFP
b.
14.3
30.4
31.5
23.7
cTnT
47.3
5.55
42.3
4.92
CD90
32.1
2.20
62.9
2.85
CD31
7.58
0.37
90.9
1.12
CD45
54.6
0.17
41.0
4.28
Ter119
Foxa2Cre:YFP

Supplementary Figure 13: Contribution of Foxa2-vCPs to the differentiated lineages in the heart. (a-b) Flow cytometry analysis of E13.5 atrial (a) and ventricular (b) chambers for cardiomyocytes (cTnT), endothelial cells (CD31), mesenchymal/epicardial cells (CD90) and hematopoietic lineages (CD45, Ter119). Green numbers highlight the number of cells that express YFP and are therefore derived from Foxa2-vCPs. **(c)** IF analysis of E15.5 *Foxa2Cre:YFP* heart cryosections showing that YFP labels Wt1+Endoglin- epicardial cells. Arrowheads indicate cells that express Wt1 (red), Wt1 and Endoglin (magenta), or that co-express YFP and Wt1 (white).

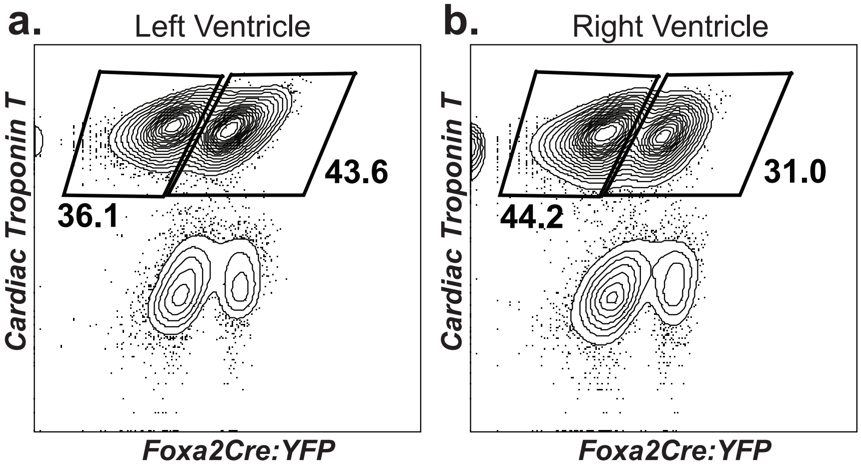
a.
Left Ventricle
36.1
43.6
Cardiac Troponin T
Foxa2Cre:YFP
b.
Right Ventricle
44.2
31.0
Cardiac Troponin T
Foxa2Cre:YFP

Supplementary Figure 14: Contribution of Foxa2-vCPs to the four-chambered heart. (a-b) Flow cytometric analysis of subdissected E13.5 *Foxa2Cre:YFP* hearts labeled with antibodies against cTnT showing that similar numbers of cardiomyocytes are derived from Foxa2-vCPs in both the left (a) and right (b) ventricle.

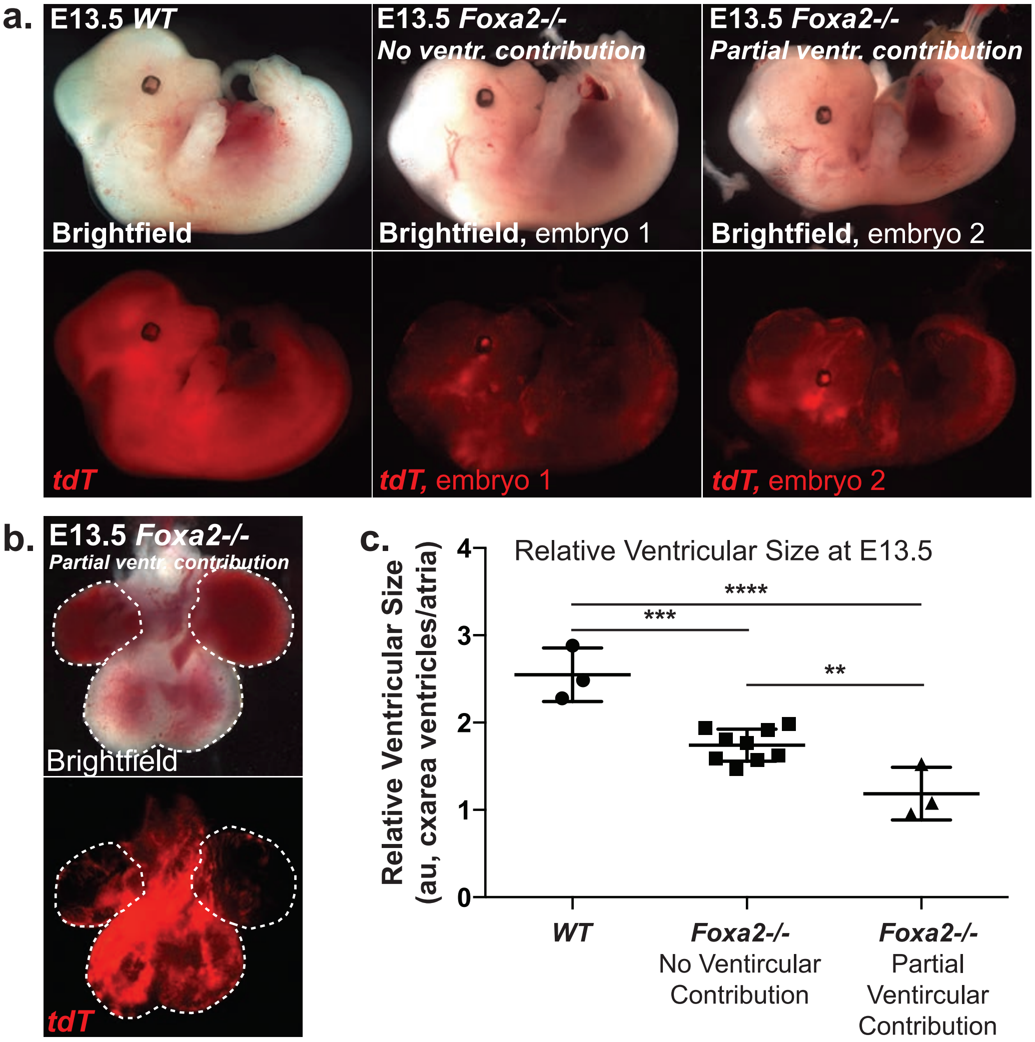
a.
E13.5 WT
Brightfield
tdT
E13.5 Foxa2-/-
No ventr. contribution
Brightfield, embryo 1
tdT, embryo 1
E13.5 Foxa2-/-
Partial ventr. contribution
Brightfield, embryo 2
tdT, embryo 2
b.
E13.5 Foxa2-/-
Partial ventr. contribution
Brightfield
tdT
c.
Relative Ventricular Size at E13.5
Relative Ventricular Size
(au, cxarea ventricles/atria)
0
1
2
3
4
****
***
**
WT
Foxa2-/-
No Ventircular
Contribution
Foxa2-/-
Partial
Ventircular
Contribution

Supplementary Figure 15: Assessment of the role of Foxa2 for cardiac development. (a) Whole mount imaging of chimeric embryos injected with wt (left) and Foxa2-/- (middle and right) mESCs. Middle panels show embryos with no contribution of Foxa2-/- cells to the ventricles; right panels show embryos with partial contribution of Foxa2-/- cells to the ventricles. **(b)** Whole mount imaging of representative hearts with partial contribution of Foxa2-/- cells to the ventricles. **(c)** Quantification of relative ventricular size in wt vs Foxa2-/- mESC injected hearts. Data is mean ± SD. Note that the ventricular size is significantly reduced when Foxa2-/- cells contribute to ventricular areas.
